# A comparison of transcriptome analysis methods with reference genome

**DOI:** 10.1186/s12864-022-08465-0

**Published:** 2022-03-25

**Authors:** Xu Liu, Jialu Zhao, Liting Xue, Tian Zhao, Wei Ding, Yuying Han, Haihong Ye

**Affiliations:** 1 Department of Medical Genetics and Developmental Biology, School of Basic Medical Sciences, Capital Medical University, Beijing, China; 2 Beijing Key Laboratory of Neural Regeneration and Repair, Capital Medical University, Beijing, China; 3 Monogenic Disease Research Center for Neurological Disorders, Beijing Tiantan Hospital, Capital Medical University, Beijing, China; 4 China National Clinical Research Center for Neurological Diseases, Beijing, China

**Keywords:** Transcriptome data analysis, RNA-seq, Differentially expressed analysis, Differentially expressed genes (DEGs), *DESeq2*, *Ballgown*, *Cuffdiff*, *Sleuth*

## Abstract

**Background:**

The application of RNA-seq technology has become more extensive and the number of analysis procedures available has increased over the past years. Selecting an appropriate workflow has become an important issue for researchers in the field.

**Methods:**

In our study, six popular analytical procedures/pipeline were compared using four RNA-seq datasets from mouse, human, rat, and macaque, respectively. The gene expression value, fold change of gene expression, and statistical significance were evaluated to compare the similarities and differences among the six procedures. qRT-PCR was performed to validate the differentially expressed genes (DEGs) from all six procedures.

**Results:**

*Cufflinks*-*Cuffdiff* demands the highest computing resources and *Kallisto*-*Sleuth* demands the least. Gene expression values, fold change, *p* and *q* values of differential expression (DE) analysis are highly correlated among procedures using *HTseq* for quantification. For genes with medium expression abundance, the expression values determined using the different procedures were similar. Major differences in expression values come from genes with particularly high or low expression levels. *HISAT2*-*StringTie*-*Ballgown* is more sensitive to genes with low expression levels, while *Kallisto*-*Sleuth* may only be useful to evaluate genes with medium to high abundance. When the same thresholds for fold change and *p* value are chosen in DE analysis, *StringTie*-*Ballgown* produce the least number of DEGs, while *HTseq*-*DESeq2*, -*edgeR* or -*limma* generally produces more DEGs. The performance of *Cufflinks*-*Cuffdiff* and *Kallisto*-*Sleuth* varies in different datasets. For DEGs with medium expression levels, the biological verification rates were similar among all procedures.

**Conclusion:**

Results are highly correlated among RNA-seq analysis procedures using *HTseq* for quantification. Difference in gene expression values mainly come from genes with particularly high or low expression levels. Moreover, biological validation rates of DEGs from all six procedures were similar for genes with medium expression levels. Investigators can choose analytical procedures according to their available computer resources, or whether genes of high or low expression levels are of interest. If computer resources are abundant, one can utilize multiple procedures to obtain the intersection of results to get the most reliable DEGs, or to obtain a combination of results to get a more comprehensive DE profile for transcriptomes.

**Supplementary Information:**

The online version contains supplementary material available at 10.1186/s12864-022-08465-0.

## Background

In recent years, RNA sequencing (RNA-seq) technology has developed rapidly, enabling the analysis of differential expression for transcriptomes in many fields. As the application has become more widespread, the number of software programs used for RNA-seq analysis has increased. Hundreds of programs, each with unique characteristics and applications, are used by researchers world-wide [1]. While experienced researchers may have good understanding of the available software and have their personal application preferences, for many researchers, especially those new to the field, choosing the appropriate software for analysis could be challenging. The existence of many analytical procedures provides more options for researchers and the appropriate software may be chosen based on the scientific problems to be solved as well as the computing resources available [2].

**Fig. 1:**
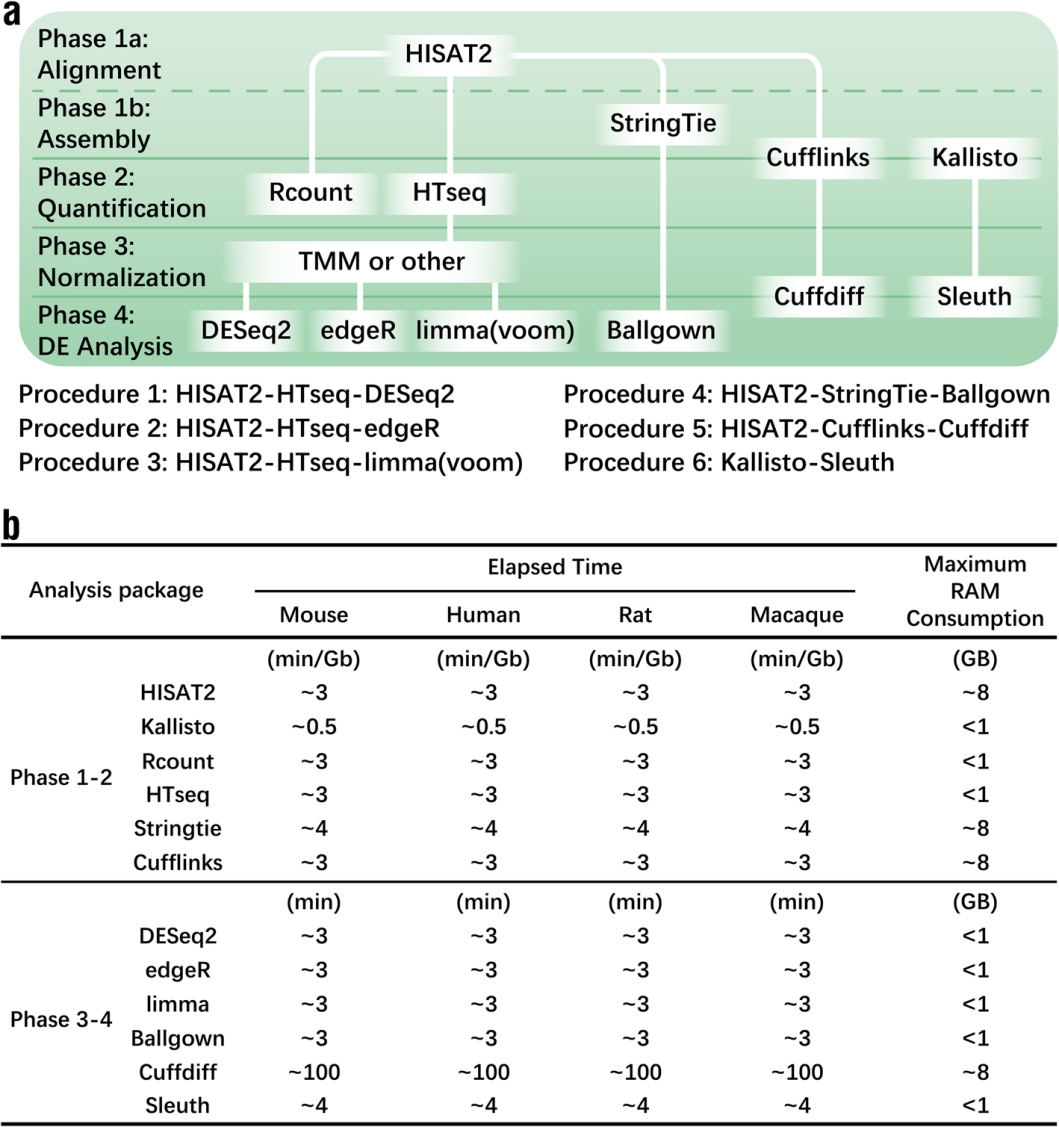
A schematic overview of the evaluation workflow. **a** The six procedures for RNA-seq analysis compared in this article are as follows: (1) *HISAT2-HTseq-DESeq2*; (2) *HISAT2-HTseq-edgeR*; (3) *HISAT2-HTseq-limma*; (4) *HISAT2-StringTie-Ballgown*; (5) *HISAT2-Cufflinks-Cuffdiff*; (6) *Kallisto-Sleuth*. **b** Time and memories consumed by each software

RNA-seq analytical procedures can be classified into two categories: reference and non-reference genome analyses. Regardless of the procedure selected, four phases, alignment and assembly, quantification, normalization, differential expression (DE) analysis, are generally required to determine the differentially expressed genes (DEGs) between two groups of samples (Fig. 1a) [3]. The software employed and the input files required for each of four phases differ according to the analytical procedure (Fig. 1a).

Phase 1, the alignment and assembly phase, requires data files in the FASTQ format [4] containing the raw sequenced reads. The most popular alignment tools used in this phase are *TopHat* [5], *HISAT* [6], and *STAR* [7], all of which require a reference genome. *HISAT* is a widely used program at present and it is an advanced version of *TopHat*. It also requires fewer computing resources than *STAR*. All these programs utilize their own algorithms to cut and align the reads to adjacent exons in the reference genome to improve the mapping rate. In some cases, the original reads may be spliced and associated with software-constructed transcriptomes to improve the alignment. The tools used for these procedures, including *StringTie* [8] and *Cufflinks* [9], can detect de novo transcripts. Moreover, when the annotation for the reference genome is incomplete, these tools can effectively fill the gap for the missing annotation information.

All the above software tools used in Phase 1 are based on an earlier concept of RNA-seq analysis, which involves first aligning raw sequencing reads to a reference genome and then establishing an association between the raw sequencing reads and the transcript. Several research teams recently have introduced pseudo-alignment or “alignment-free” tools. These tools, including *Kallisto* [10] (Fig. 1a) and *Salmon* [11], can directly associate the raw sequencing reads with the transcript and evaluate the gene or transcript expression levels. These processes are generally carried out in Phase 2 for the mainstream analysis procedures.

Phase 2 in RNA-seq analysis involves evaluating the expression level of genes or transcripts according to the sequencing reads aligned to the reference genome in Phase 1. Previous studies have shown that quantification tools have a greater impact on the final DE results than alignment tools [12, 13]. Commonly used quantification tools include *Rcount* [14], *HTseq* [15], *StringTie* [8], and *Cufflinks* [9] (Fig. 1a). These tools can be divided into two groups according to the evaluation standards for gene expression which can be based on counts or fragments per kilobase of transcript per million mapped reads (FPKM) values. *Rcount*, *HTseq*, and *Kallisto* are based on counts, while *StringTie* and *Cufflinks* are based on FPKM values. Both *HTseq* and *Rcount* count the reads mapped unambiguously to a single gene. *HTseq* discards the reads aligned to multiple positions and those that overlap with more than one gene [15], while *Rcount* assigns weights to each alignment of a multiread [14]. Therefore, *Rcount* is better at counting multireads and gene overlapping regions. Generally, when the reads in a dataset have good quality and length, unambiguous reads account for the majority of the transcriptome. *StringTie* and *Cufflinks* were developed by the same research team [8, 9]. Both quantify the gene expression levels based on FPKM values. The expression values for different transcripts can be determined from the results of these two programs. The resulting values for different transcripts of the same gene can be combined to obtain the gene expression values for DE analysis.

Most analysis procedures used to evaluate gene expression based on counts require a third phase, in which an expression matrix is constructed using quartile or median normalization methods [16, 17] (Fig. 1a). Once an expression matrix is constructed, a matrix of expression values can be modeled to determine which gene or transcript features are likely to have altered expression levels. Earlier studies have shown that the normalization methods used in Phase 3 may have a major impact on the results of DE analysis [18, 19]. Procedures used to evaluate gene expression based on FPKM values [20] do not require a third phase; however, the normalization methods may be slightly inadequate in explaining the guanine-cytosine (GC) content [21] and read depth [22]. Software tools used for DE analysis in Phase 4 include *DESeq2*[23], *edgeR* [24], *limma* [25], *Ballgown* [26], *Cuffdiff* [9], and *Sleuth* [27].

Different combinations of analytical tools at these four phases generate hundreds of alternative procedures/pipelines for RNA-seq analysis. Therefore, the major challenge in this field is for users to choose between many possible methodological options according to their needs and to obtain accurate results. Many possible combinations of tools have been comparatively analyzed to date, but their performance remains under discussion [1, 12, 28, 29]. Corchete et al. performed a thorough comparison of 192 pipelines applied to 18 samples of human cell lines. Based on the precision and accuracy of raw gene expression quantification and DEG detection, they provided a guide to the different procedures for RNA-seq analysis [30].

Here we investigated the differences and characteristics of the results obtained with six analytical procedures that are most commonly used for RNA-seq analysis to date (Fig. 1a) [3]. We compared five different quantification tools, specifically *Rcount*, *HTseq*, *StringTie*, *Cufflinks*, and *Kallisto*, and six different tools for DE analysis, namely *DESeq2*, *edgeR*, *limma*, *Ballgown*, *Cuffdiff*, and *Sleuth*. The six analysis procedures were: (1) *HISAT2*-*HTseq*-*DESeq2*; (2) *HISAT2*-*HTseq*-*edgeR*; (3) *HISAT2*-*HTseq*-*limma*; (4) *HISAT2*-*StringTie*-*Ballgown*; (5) *HISAT2*-*Cufflinks*-*Cuffdiff*; (6) *Kallisto*-*Sleuth*. All six procedures were applied to RNA-seq datasets from four different organisms (mouse, human, rat, and macaque) to make the results more convincing. Our goal is to help researchers determine the optimal analytical procedure for their needs in terms of the computing resources available, time consumption, and their research goals.

## Methods

### Computing resource and software operating environment

All analyses were performed on the same computer equipped with a Microsoft Windows 10 system, 64 GB random access memory (RAM), and an Intel Core i9-9900 K CPU. The programs that required a Linux system were installed in a virtual machine in VirtualBox, and the operating system on the virtual machine was Bio-Linux 8.0.7.

### Data collection and quality control

An RNA-seq dataset from samples of mouse (*Mus musculus*) prefrontal cortices was obtained based on previous work in our laboratory (NCBI, GSE111708) [31]. FASTQ and metadata files from an RNA-seq dataset from samples of human (*Homo sapiens*) lymphoblastoid cell lines were obtained from the Gene Expression Omnibus (ERP001942) [32–34]. The RNA-seq datasets from samples of rat (*Rattus norvegicus*) lung tissues and macaque (*Macaca mulatta*) blood were obtained from GSE159668 [35] and GSE184949 [36] of the Gene Expression Omnibus database, respectively. After downloading and decompressing the datasets, the integrity of the data was checked and quality control was performed with *FastQC* (version 2.11.5). The total number of samples and the data sizes for individual samples are presented in Supplementary Table 1.

### Alignment

Alignment software can be divided into two types: accurate alignment and pseudo-alignment (Fig. 1a). The reference genomes used were *Mus musculus* GRCm38 for mouse datasets, *Homo sapiens* GRCh38 for human datasets, *Rattus norvegicus* Rnor6 for rat datasets, and *Macaca mulatta* Mmul10 for macaque datasets. Accurate alignment was executed using *HISAT2* (version 2.1.0). Pseudo-alignment was performed using *Kallisto* (version 0.46.1). For *Kallisto*, the mapping rate was calculated as the proportion of the pseudo-aligned reads to the total reads.

### Assembly and quantification

Five different assembly and quantification software packages were used: *Rcount*, *HTseq*, *StringTie* (version 1.3.4d), *Cufflinks* (version 2.2.1), and *Kallisto* (Fig. 1a). *Samtools* (version 1.9) was used for the file format conversion required during the alignment and quantification steps.

### Differential expression analysis

Tools that perform DE analysis include *DESeq2* (version 1.22.2), *edgeR* (version 3.24.3), *limma* (version 3.38.3), *Ballgown* (version 2.14.1), *Cuffdiff* (version 2.2.1), and *Sleuth* (version 3.30.3). *Cuffdiff* was installed in Bio-Linux 8.0.7. Other tools were run in the *R environment* (R-3.6.3). *DESeq2*, *edgeR*, and the *limma* package used the trimmed mean for M values (*TMM*) for expression normalization.

### Correlation analysis

Correlation analysis was performed to compare gene expression values, fold changes (FCs), and statistical significance. Correlation analysis was performed using the cor package in the *R environment* (R-3.6.3). Pearson correlation coefficient was calculated to evaluate the correlation of pairwise comparisons. During the comparison process, inconsistencies in the number of results were inevitable; that is, some genes only appeared in the results from one procedure. In these situations, only the expressed genes identified in both procedures were retained. We defined the top 10% of all retained genes as high expression genes, the bottom 10% as low expression genes, and the 80% in between as medium abundance genes.

### Quantitative real-time polymerase chain reaction

Total RNA was extracted from the mouse prefrontal cortices (PFCs) using TRIzol reagent (Invitrogen) and the messenger RNAs (mRNAs) were subsequently extracted. A complementary DNA (cDNA) Synthesis Kit (New England Biosystems) was used to synthesize cDNA. Quantitative real-time polymerase chain reaction (qRT-PCR) was performed using the cDNA Synthesis Kit (Kapa Biosystems). The sequences for the primers (synthesized in Invitrogen) are listed in Supplementary Table 2. Quantification of gene expression was performed in a DNA thermocycler (CFX Connect, Bio-Rad) using a three-step cycling protocol. The housekeeping gene *Gapdh* was used as an endogenous control to normalize the mRNA content in each sample. Normalized mRNA levels were quantified using the comparative C(T) method. A DEG was confirmed only when FC of mRNA level and *p* value from qRT-PCR met the criteria that |log_2_FC|> 1 and *p* < 0.01. Verification rates (VR) were defined as the number of genes that passed validation divided by the total number of genes that were assessed using qRT-PCR.

### Statistical analysis

Statistical analysis was performed using the GraphPad Prism or R packages. All data are represented as the mean ± the standard error of the mean (SEM). The statistical significance values for all bioinformatic analyses are presented in the results.

## Results

### Comparison of the computing resources consumed by different procedures

To compare the computing resources consumed by different procedures during different phases for different species, we performed the same data analysis on four sets of RNA-seq data from four model organisms, including mouse [31], human [32, 34], rat [35], and macaque [36]. The total number of samples and the data size of individual samples were presented in Supplementary Table 1. Data analysis was performed using our in-house computers with a Microsoft Windows 10 system, 64 GB RAM, and an Intel Core i9-9900 K CPU. The analysis procedures and software used are listed in Fig. 1a and the time consumption for different procedures is shown in Fig. 1b.

On our in-house computers, the time required by the same software for these four different RNA-seq datasets was roughly the same, suggesting that when the sizes of the reference genome are comparable, the computational resources consumed are practically equal. Comparing the time required by different analytical procedures, we found that *Kallisto* exhibited the fastest computing speed in Phases 1 and 2. In Phases 3 and 4, the time and computer memory required by *Cuffdiff* were much higher than those for other methods. These results indicate that *Kallisto*-*Sleuth* may require the least computing resources, while *Cufflinks*-*Cuffdiff* requires the most.

### Comparison of the gene expression levels determined with different quantitative methods

**Fig. 2:**
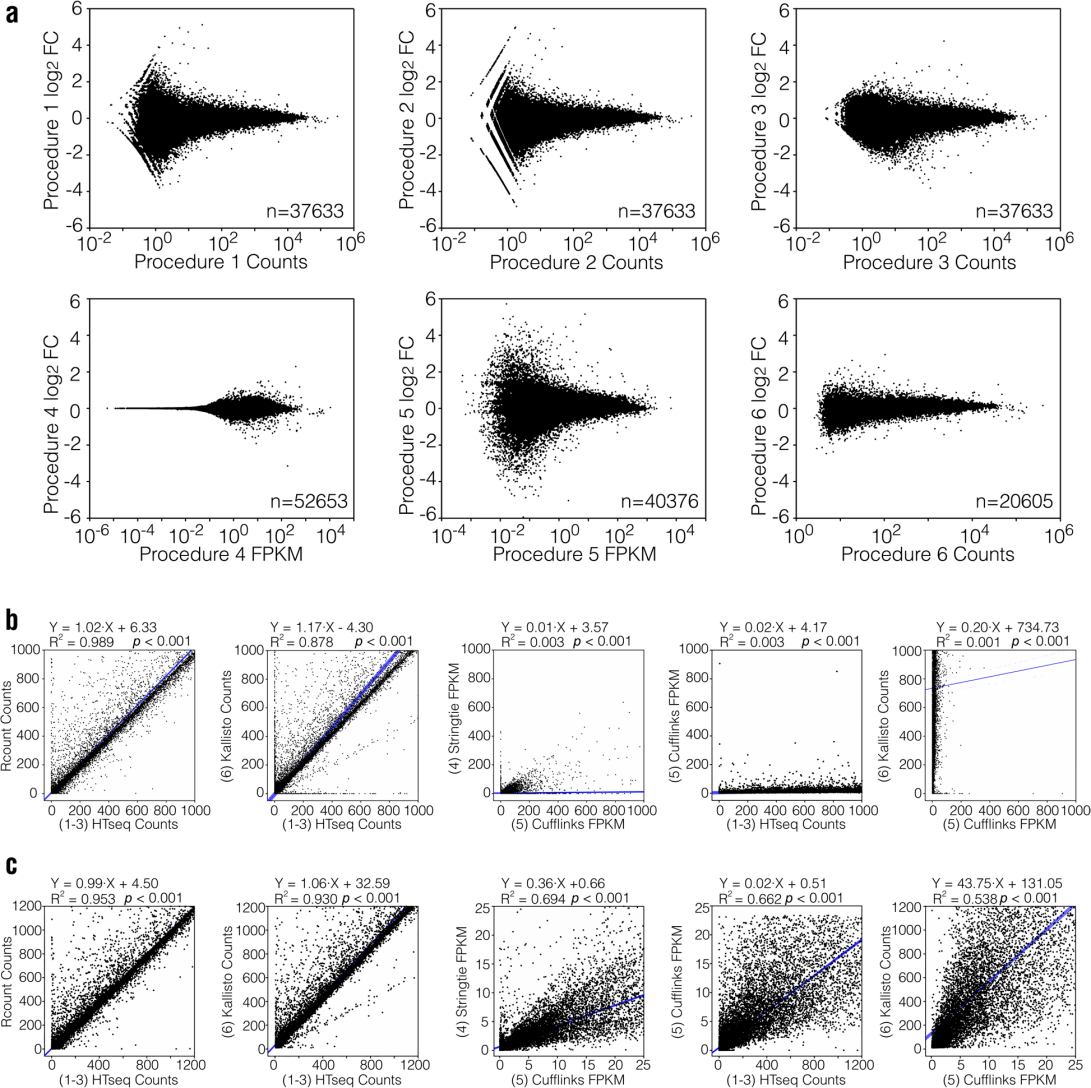
Evaluation and comparison of genes expression levels in the mouse dataset. **a** MA plots of different analytical procedures. **b** Comparison of gene expression levels evaluated by different quantitative software without any screening. **c** Comparison of gene expression levels obtained with different quantitative software after removing the genes with the top and bottom 10% expression levels. The numbers in brackets represents the procedure number. *R*^2^ was calculated via Pearson’s correlation analysis

To compare the gene expression levels determined by different procedures, we first created MA plots to illuminate the relationship between the gene expression level and FC of gene expression determined using each procedure (Fig. 2a for mouse data; Supplementary Fig. 1a, b, and c for human, rat, and macaque data, respectively). It can be seen from the MA plots that range for gene expression values determined by Procedures 1, 2, and 3 were basically the same (between 10^–2^ and 10^6^ for all four datasets). The results for Procedure 5, *Cufflinks*-*Cuffdiff*, accounted for genes with average FPKM between 10^–4^ and 10^4^. The results for Procedure 4, *StringTie*-*Ballgown*, accounted for genes with average FPKM as low as 10^–6^, while those for Procedure 6, *Kallisto*-*Sleuth*, only accounted for genes with average counts above 1. These results suggest that Procedure 4 is more sensitive to genes with low expression levels, while Procedure 6 may only be useful to evaluate genes with medium to high abundance. The MA plot can also reflect the total number of genes evaluated by different procedures Because the analytical tools used in Phase 2 of Procedure 1, 2, and 3 are the same, the total number of genes finally evaluated is the same within each dataset, but the total number of genes produced varied between different datasets. Procedure 6 yielded the least number of genes in all four datasets, which may be due to its insensitivity to genes with low expression levels. The performance of other procedures varied in different datasets (Fig. 2a for mouse data; Supplementary Fig. 1a, b, and c for human, rat, and macaque data, respectively).

We next examined the pair-wise correlation of gene expression values produced by different quantification methods. To simplify the number of comparisons, we divided quantitative tools into three groups: 1) *Rcount* and *HTseq* that evaluate gene expression values as counts; 2) *StringTie* and *Cufflinks* that use FPKM values; and 3) *Kallisto* that performs pseudo-alignment using counts. First, we compared the gene quantitative software within each group. If there was a high correlation within the group, we selected the most commonly used software within the group as the representative tool for inter-group comparison.

Upon comparing the results for these four datasets gene expression values, we found that the three Procedures that evaluated gene expression values using counts (*Rcount*, *HTseq*, and *Kallisto*) showed high correlations (Fig. 2b for mouse data; Supplementary Figs. 3, 4 and 5a, for human, rat, and macaque data, respectively). The Pearson correlation coefficients (*R*^2^) between *Rcount* and *HTseq* were higher than 0.9 and those between *Kallisto* and *HTseq* were higher than 0.8 in all four datasets. Although both *StringTie* and *Cufflinks* use FPKM values to evaluate gene expression levels, results obtained with these tools exhibited poor correlations in all four datasets (Fig. 2b for mouse data; Supplementary Figs. 3, 4 and 5a, for human, rat, and macaque data, respectively). Algorithms that use counts and FPKM were also compared and neither *HTseq* nor *Kallisto* correlated well with *Cufflinks* (Fig. 2b for mouse data; Supplementary Figs. 3, 4 and 5a, for human, rat, and macaque data, respectively).

To explore the source of differences in the gene expression levels obtained with different procedures, a logarithmic transformation was performed on the gene expression values [log_10_(gene expression level)] and the Pearson correlation analysis was repeated. The pair-wise correlation coefficients among *StringTie*, *Cufflinks*, *HTseq*, and *Kallisto* were much higher after the logarithmic transformation (Supplementary Fig. 2 for the mouse dataset; Supplementary Figs. 3, 4 and 5b for the human, rat, and macaque datasets, respectively). As logarithmic transformation generally reduces differences in very high or very low values, these results suggested that the source of differences in the gene expression levels obtained with different procedures may be due to genes with very high or very low expression levels. Indeed, when genes with the top and bottom 10% expression levels were removed, the Pearson correlation coefficients for gene expression levels (without logarithmic transformation) between *StringTie*, *Cufflinks*, *HTseq*, and *Kallisto* were increased in all four datasets (Fig. 2c for the mouse dataset and Supplementary Figs. 3, 4 and 5c for the human, rat, and macaque datasets, respectively). Together, these results indicate that the differences in gene expression obtained with different procedures mainly come from genes with particularly high or low expression levels. For genes with medium abundance, the expression levels determined using different procedures were comparable. We also found that the correlation coefficients between *HTseq* and *Rcount* were very high (Fig. 2b for the mouse dataset; Supplementary Figs. 3, 4 and 5a, for the human, rat, and macaque datasets, respectively), indicating that these two methods can be mutually substituted. Therefore, we used the results from *HTseq* to perform the subsequent normalization and DE analysis procedures for Procedures 1, 2, and 3.

### Comparison of differentially expressed genes obtained with different software

After comparing the gene expression values, we next compared FCs for gene expression levels, *p* values, and corrected *p* values for each gene between two groups of RNA-seq samples (control vs. case) obtained from the DE analysis software in Phase 3 and 4. We analyzed all these four different RNA-seq datasets using all six analytical procedures with the same parameter settings. FC values, *p* values, and corrected *p* values were extracted from the outputs of all six procedures for subsequent correlation analysis.

### Fold change

**Fig. 3:**
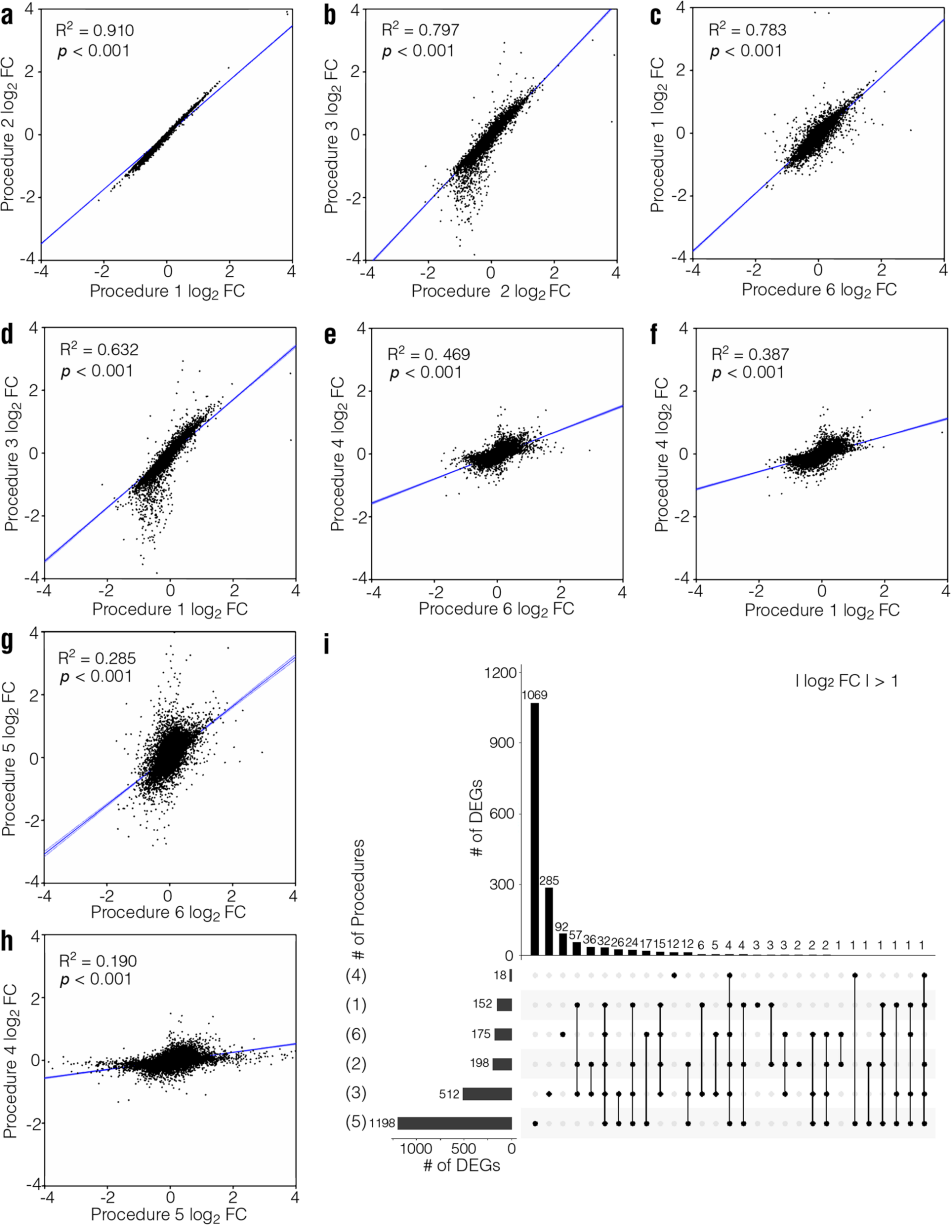
Evaluation and comparison of fold change (FC) of gene expression levels obtained with different analytical procedures for the mouse dataset. **a-h** Comparison of log_2_FC obtained with different procedures. *R*^2^ and *p* were calculated via Pearson’s correlation analysis. **i** Set visualization graphics of DEG numbers when |log_2_FC|> 1 was used as threshold to define DEGs. The numbers in brackets represent the procedure number

Firstly, FC values produced by all six analytical procedures were compared using Pearson correlation analysis. We observed high correlations among the results from Procedures 1 (*DESeq2*), 2 (*edgeR*), 3 (*limma*), and 6 (*Sleuth*) (all *R*^2^ > 0.6, Fig. 3a-d for the mouse dataset; Supplementary Figs. 6, 7 and 8a-d for the human, rat, and macaque datasets, respectively). Among them, Procedures 1 and 2 showed the highest level of correlation (*R*^2^ > 0.9), probably because *DESeq2* and *edgeR* employ the same normalization method. Procedures 4 (*Ballgown*) and 5 (*Cuffdiff*) exhibited poor correlation with each other (*R*^*2*^ = 0.190 for the mouse dataset, *R*^*2*^ = 0.466 for the human dataset, *R*^*2*^ = 0.400 for the rat dataset, and *R*^*2*^ = 0.281 for the macaque dataset, Fig. 3h and Supplementary Figs. 6, 7 and 8h). They also showed poor correlation with the other four procedures (Fig. 3e-h for the mouse dataset; Supplementary Figs. 6, 7 and 8e–h for the human, rat, and macaque datasets, respectively).

To explore the overlapping DEGs among different procedures, a threshold of |log_2_FC|> 1 was used to define the DEGs and a set of visualization graphics was created (Fig. 3i for the mouse data; Supplementary Figs. 6, 7 and 8i for the human, rat, and macaque datasets, respectively). Procedure 4 produced the least number of DEGs in the mouse, human, and rat datasets (18 for the mouse dataset, 335 for the human dataset, and 333 for the rat dataset), and very few DEGs overlapped with those obtained with the other procedures. Procedure 5 produced the highest number of DEGs (1198 for the mouse dataset, 2258 for the human dataset, 1495 for the rat dataset, 1742 for the macaque dataset), although only a small fraction overlapped with the results from the other five procedures (Fig. 3i for the mouse dataset; Supplementary Figs. 6, 7 and 8i for the human, rat, and macaque datasets, respectively). Procedures 1, 2, 3, and 6 showed high levels of overlap in DEGs, consistent with the Pearson correlation analysis.

Together, the above results indicate that FCs of gene expression levels from Procedure 1, 2, and 3 show a good correlation with each other. When using |log_2_FC|> 1 as the threshold, Procedure 4 generally produces the least number of genes, while Procedure 5 yields the highest number of genes.

### *p* values and corrected *p* values (*q* values)

**Fig. 4:**
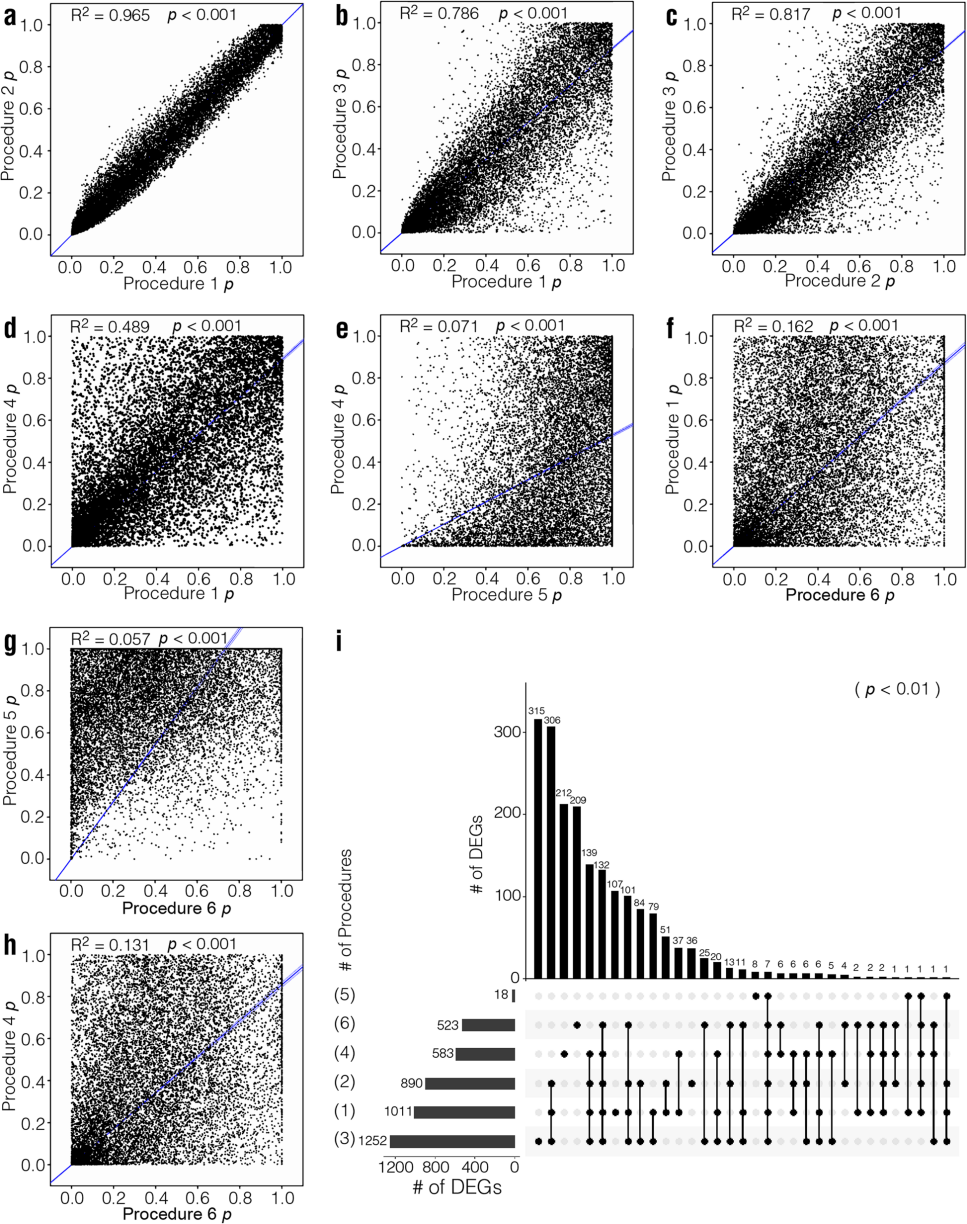
Evaluation and comparison of *p* values from different analytical procedures for the mouse dataset. **a-h** Comparison of *p* values obtained from different procedures. *R*^2^ and *p* were calculated via Pearson’s correlation analysis. **i** Set visualization graphics of DEG numbers when *p* < 0.01 was used as threshold to define DEGs. The numbers in brackets represent the procedure number

*p* values and corrected *p* values produced by different procedures were also compared using Pearson correlation analysis (Fig. 4 for the mouse dataset, Supplementary Figs. 9, 10 and 11 for the human, rat, and macaque datasets, respectively). *p* values in the Pearson correlation analysis from Procedures 1 and 2 exhibited the highest correlation (*R*^*2*^ = 0.965 for the mouse dataset, *R*^*2*^ = 0.961 for the human dataset, *R*^*2*^ = 0.975 for the rat dataset, *R*^*2*^ = 0.960 for the macaque dataset). *p* values from Procedure 3 also correlated well with those from Procedures 1 and 2 (*R*^*2*^ > 0.78 for the mouse dataset, *R*^*2*^ > 0.85 for the human dataset, *R*^*2*^ > 0.91 for the rat dataset, *R*^*2*^ > 0.88 for the macaque dataset), most likely because the normalization methods employed by these three procedures were the same. In contrast, *p* values from Procedures 4, 5, and 6 correlated poorly with those from other procedures, as the basic statistical models used in these three Procedures for DEG analysis were fundamentally different. Consistent with these results, when *p* < 0.01 was used as the threshold to define DEGs, the number of overlapping DEGs from Procedures 1, 2, and 3 was high, while those from the other Procedures were relatively low (Fig. 4i for the mouse dataset; Supplementary Figs. 9, 10 and 11i for the human, rat, and macaque datasets, respectively).

As *p* value correction is necessary for multiple statistical testing, *q* value is widely used to set a threshold for defining DEGs in RNA-seq analysis. Therefore, we also performed correlation analysis with *q* values and the results were consistent with those for *p* values in the mouse and the human datasets. In the rat and the macaque datasets, the correlation between Procedures 1 and 2 was also high, but Procedures 2 and 3 have the highest correlation (Supplementary Figs. 12–15, for the mouse, human, rat, and macaque datasets, respectively). When DEGs were defined as genes with *q* < 0.05, Procedures 1 produced the highest number of DEGs in the mouse, the human, and the macaque datasets, and the second-highest in the rat dataset. The performance of other procedures varied considerably. Some procedures yielded an insufficient number of DEGs for further analysis in certain datasets (Supplementary Figs. 12–15).

Together, the above results indicate that *p* values and *q* values correlated well among Procedure 1, 2, and 3, which is consistent with Corchete et al.’s report [30]. When using *q* < 0.05 as the threshold, Procedures 1 generally produced the highest number of DEGs.

### Number of differentially expressed genes produced by different procedures

As a general practice in the field, DEGs are defined taking into consideration both the FC of gene expression levels and the *p* or *q* value in statistical analysis. Therefore, we defined DEGs with both criteria and compared the results of DE analysis with different procedures.

**Fig. 5:**
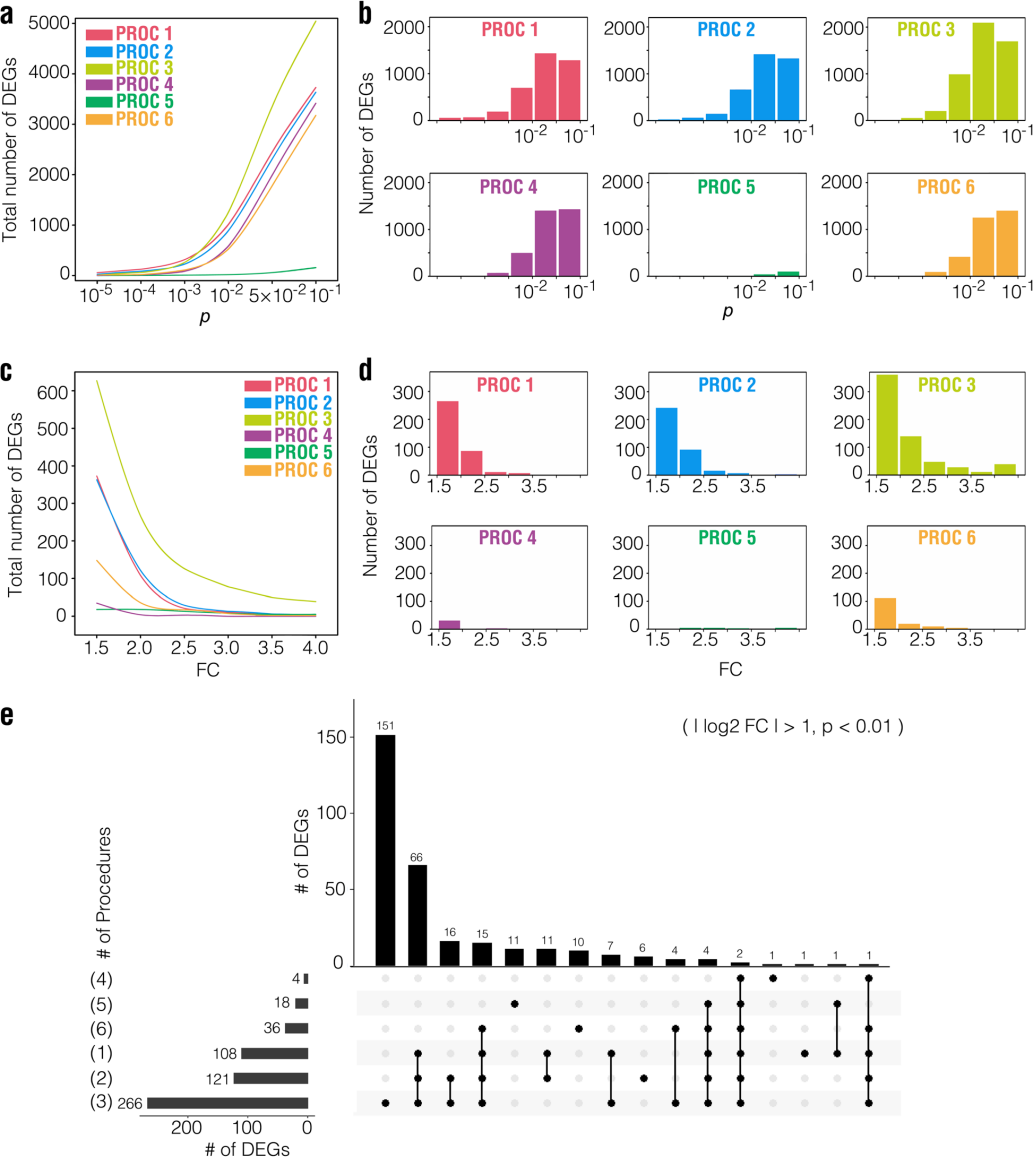
Number of DEGs defined with combination of FC and *p* value for the mouse dataset. **a** The line chart reflects the total number of DEGs estimated by different procedures with |log_2_FC|> 1 and different *p* values. **b** The histogram reflects the interval number of DEGs estimated by different procedures with |log_2_FC|> 1 and different *p* values. **c** The line chart reflects the total number of DEGs estimated by different procedures with *p* < 0.01 and different |log_2_FC|. **d** The histogram reflects the interval number of DEGs estimated by different procedures with *p* < 0.01 and different |log_2_FC|. **e** Set visualization graphics of DEG numbers when |log_2_FC|> 1 and *p* < 0.01. The numbers in brackets represent the procedure number

When the threshold for FC was set as |log_2_FC|> 1 in the mouse data analysis, the number of DEGs increased rapidly with the increasing *p* value when *p* is above 0.01 for all procedures except Procedure 5 (Fig. 5a and b). The situations for the other three datasets were similar, except that Procedure 5 also produced a considerable number of DEGs when *p* > 0.01 (Supplementary Figs. 16, 17 and 18a and b, for the human, rat, and macaque datasets, respectively), indicating that the threshold may vary for different RNA-seq datasets.

When using *p* < 0.01 as the statistical threshold, a considerable number of DEGs were obtained with FCs between 1.5 and 4.5 using Procedures 1, 2, 3, and 6, but very few were obtained with Procedures 4 and 5 for the mouse dataset (Fig. 5c and d). Procedure 4 still produced very few DEGs for the human and the rat datasets, while Procedure 5 exhibited better performance in the human, rat, and macaque datasets (Supplementary Figs. 16, 17 and 18c and d, for the human, rat, and macaque datasets, respectively). When |log_2_FC|> 1 and *p* < 0.01 were set as the threshold for the mouse dataset, 88 overlapping DEGs were obtained with Procedures 1, 2, and 3, but much fewer overlapping DEGs were obtained with the other procedures (Fig. 5e). Only two overlapping DEGs were obtained with all procedures (Fig. 5e). In the human dataset, 427 DEGs were shared among Procedures 1, 2, and 3, whereas 45 DEGs were obtained with all procedures (Supplementary Fig. 16e). Procedures 1, 2, and 3 also produced the highest number of overlapping DEGs in the rat and the macaque datasets, whereas 7 and 27 DEGs were obtained with all procedures in the rat and the macaque datasets, respectively (Supplementary Figs. 17 and 18e).

When |log_2_FC|> 1 and *q* < 0.05 were set as the thresholds for DEGs in the mouse dataset, Procedures 1, 2, and 3 still produced a sufficient number of DEGs, but very few or no DEGs were obtained with the other procedures (Supplementary Fig. 19). When the same thresholds were applied to the human dataset, all procedures, except Procedure 4, produced a considerable number of DEGs (Supplementary Fig. 20). When the same thresholds were applied to the rat and the macaque datasets, Procedures 1 and 5 produced a sufficient number of DEG, but very few or no DEGs were obtained with the other procedures (Supplementary Fig. 21 for the rat dataset and Supplementary Fig. 22 for the macaque dataset).

Together, these results indicate that when considering both the FC of gene expression levels and the *p* (or *q*) value in defining DEGs, Procedures 4 produce the least number of DEGs, while Procedures 1, 2, and 3 generally produce more DEGs. The performance of Procedure 5 and 6 varies in different datasets. Therefore, the analytical procedures and thresholds for screening DEGs should be carefully selected according to the characteristics of the datasets.

### Verification of DEGs using qRT-PCR

**Fig. 6:**
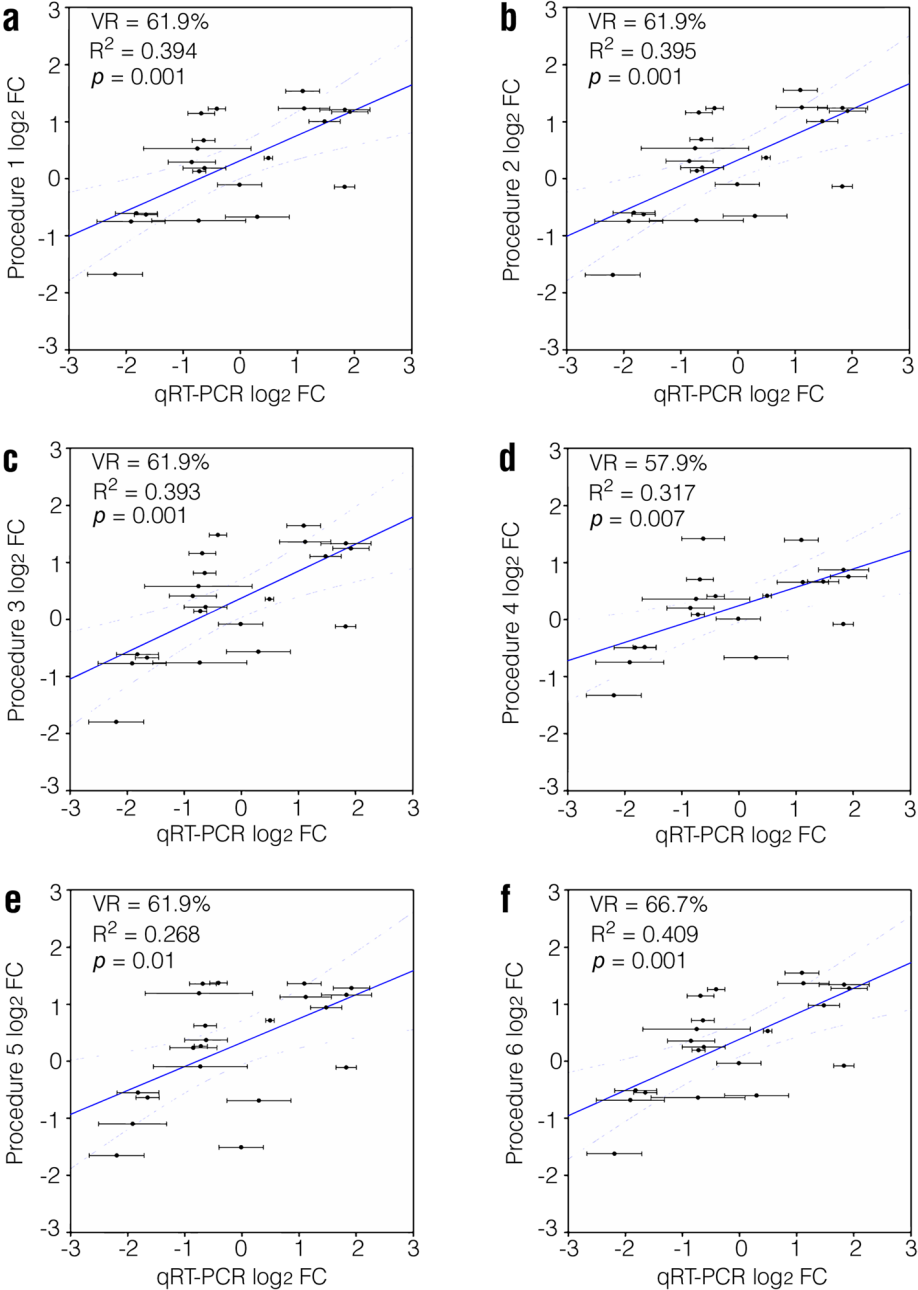
Correlation of log_2_FC for the same genes in different procedures and qRT-PCR experiments. A total of 21 genes were assessed. VR, verification rate. *R*^2^ and *p* was calculated via Pearson’s correlation analysis

Finally, we assessed the validity of DEGs obtained with the six analytical procedures. Twenty-one genes in the mouse dataset were selected for qRT-PCR analysis (Supplementary Fig. 23). These genes were DEGs from at least one procedure when |log_2_FC|> 1 and *p* < 0.01 were used to define DEGs. These genes exhibited medium expression levels, which are more likely to be relevant for biological functions and are easy for qRT-PCR analysis. The correlation coefficients between the results of qRT-PCR and the log_2_FC values from the RNA-seq analysis were similar (*R*^2^ = 0.3). When both FC of mRNA levels (|log_2_FC|> 1) and *p* value (*p* < 0.01) from qRT-PCR were considered in defining positively-validated DEGs, the verification rates (VR) were comparable (~ 60%) for all six procedures (Fig. 6). These results indicate that the performance of all six procedures in predicting verifiable DEGs is comparable for genes with medium expression levels.

## Discussion

In this study, we evaluated the performance of six commonly used differential expression analysis procedures on four datasets from mouse, human, rat, and macaque, respectively. We mainly compared the following three aspects: the computing resources and time consumed by different procedures, the quantitative values of gene expression (expression level, fold change of expression, *p* value, and *q* value) obtain in different procedures, and the validation rate of DEGs obtained through different procedures. Knowing the pros and cons of different procedures in these three aspects, one can choose a more suitable procedure for his research.

## Differences in computing resources consumed by different procedures

We assessed several indicators of computing resource consumption, including computer memory usage and time spent. Each DE analysis procedure has a minimum requirement for computer hardware and the amount of memory required for the calculation is probably the most important constraint. Before conducting DE analysis, a reference genome index file needs to be established, which usually takes more computing resources than the DE analysis itself. The index files for human or model organism genomes can also be downloaded from websites associated with the analysis software. In the four phases of DE analysis, Phase 1a usually takes up a large amount of RAM, which is determined mainly by the size of the reference genome used for alignment. According to the results of our comparison, Phases 1 and 4 required the longest duration. In general, Procedure 6 required the least RAM to complete the analysis in the shortest time, while Procedure 5 required more RAM and time than the other procedures. Interestingly, when analyzing data on personal computers, we found that a solid-state drive could significantly increase the calculation speed for all six procedures, indicating that the speed-limiting step is the reading and writing process for the hard disk when the computer has a high-performance CPU and ample RAM.

## The quantitative values of genes obtained in different procedures

In a complete DE analysis procedure, we obtained four values for quantitative analysis, namely gene expression level, FC of gene expression, *p* value, and *q* value. DEGs are selected based on these four values for further bioinformatic analysis and biological verification.

Common ways to quantify and normalize gene expression levels include the reads per kilobase of transcript per million mapped reads (RPKM) in single-ended sequencing, FPKM and transcripts per million (TPM) in pair-ended sequencing, and the count values used in both types of sequencing. In our study, different procedures were divided into a count group (Procedures 1, 2, 3, and 6) and an FPKM group (Procedures 4 and 5). Gene expression values were highly correlated among Procedures 1, 2, and 3, but poorly with the other 3 procedures. This is probably due to differences in quantification and normalization methods, as suggested by Corchete et al. [30]. However, we found that the expression values produced by the six procedures were well correlated for genes with medium expression levels. The differences among procedures were mainly found in genes with extremely high or low expression levels (Fig. 2 and Supplementary Figs. 3, 4 and 5), suggesting that RNA-seq results for genes with medium abundance are more reliable for further biological study.

FC of gene expression is an important parameter for screening DEGs. Generally, a higher FC of gene expression levels indicates more important biological functions and easier biological verification. In our study, we found that the normalization method affects the FCs obtained with different procedures, consistent with previous reports on RNA-seq analysis [12, 13]. FCs of gene expression produced by Procedures 1, 2, and 3 were highly consistent, same as the gene expression values (Fig. 3a-d and Supplementary Figs. 6, 7 and 8a-d). Procedures 4, 5, and 6 employed very different quantification and normalization algorithms and therefore produced very different results for FCs (Fig. 3e-h and Supplementary Figs. 6, 7 and 8e–h).

The *p* and *q* values are statistical values obtained in Phase 4 that indicate whether the DE is statistically significant. To some extent, these values may also reflect whether the target gene expression is stable within each group of samples compared (control group vs. case group). The *p* value is determined by the underlying statistical models used in Phase 4 for different procedures, and the *q* value is determined from the *p* value as well as the total sample size. In this study, we found that the correlation of the *p* and *q* values between Procedures 1, 2, and 3 was very high (Fig. 4a-c and Supplementary Figs. 9, 10 and 11a-c), consistent with Corchete et al.’s report [30]. The highest correlation was observed between Procedures 1 and 2 (Fig. 4a and Supplementary Figs. 9, 10 and 11a), as both procedures use negative binomial distribution as the mathematical model in Phase 4 of statistical analysis [23, 24].

Together, our results indicate that the results of gene expression levels and DEGs obtained with Procedures 1, 2, and 3 are highly similar but are different from those obtained via Procedures 4, 5, and 6. When both the FC of gene expression and the *p* (or *q*) value for defining DEGs were considered, Procedures 4 usually produce the least number of DEGs, while Procedures 1, 2, and 3 generally produced more DEGs (Fig. 5 and Supplementary Figs. 16, 17 and 18).

### Validation rates of DEGs obtained using different procedures.

In this section, we assessed the validation rates for DEGs obtained through different procedures using qRT-PCR. Genes with medium expression levels were selected as their expression can be easily assessed using qRT-PCR analysis and are more likely to be functionally relevant. Our results indicated that the validation rates for different procedures were similar (Fig. 6), indicating that the performance of all six procedures in predicting verifiable DEGs was comparable for genes with medium expression levels.

### Characteristics of different procedure and procedure selection guidance

**Fig. 7:**
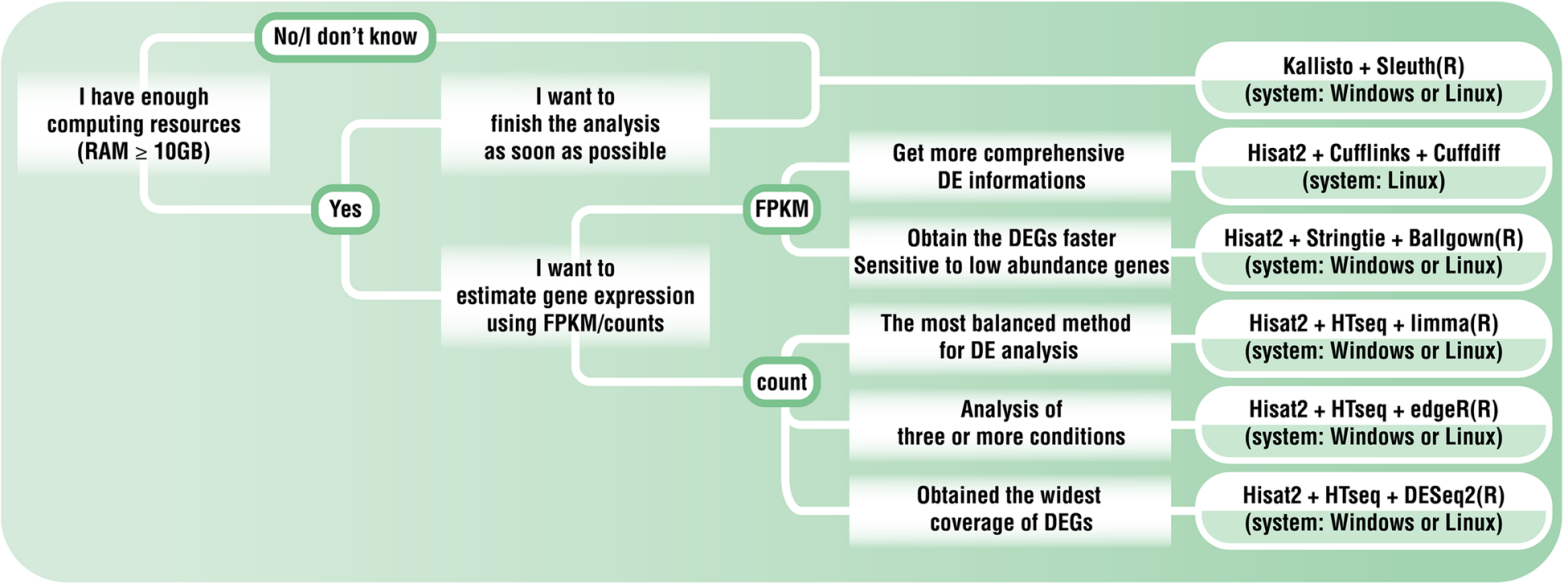
Guidelines for researchers to decide the appropriate procedure for RNA-seq analysis

Based on the above results, we summarize the characteristics and application of the six procedures in Fig. 7. If one has very limited computing resources, Procedure 6 is recommended as it consumes the least computing resources. In this study, we analyzed two quantified gene expression indicators: count and FPKM value. Users can select different quantified gene expression indicators according to their own research needs. If users prefer FPKM, Procedures 4 and 5 are more suitable. They can also provide information about de novo transcripts. Procedure 4 is more sensitive to low-expressing genes. If users prefer to use count to quantify gene expression, Procedures 1, 2, and 3 are more suitable. Results from Procedure 1, 2, and 3 are highly correlated and generally produce more DEGs than the other three procedures. In three out of the four datasets (human, rat, and macaque), Procedure 1 produced the highest number of DEGs. Coechete et al. suggested that *limma trend* is the most balanced method in accuracy and efficiency among 17 DE analysis methods, including *DEseq2* and *edgeR* [30]. Investigators with ample computing resources can use multiple procedures according to their needs and take advantage of the intersection of the analysis results to obtain the most reliable DEGs or use a combination of procedures to obtain a more comprehensive DE profile for the transcriptome.

## Conclusion

Results are highly correlated among RNA-seq analysis Procedures 1, 2, and 3, all of which use *HTseq* for quantification. The difference in gene expression values mainly come from genes with particularly high or low expression levels. Moreover, biological validation rates of DEGs from all six procedures were similar for genes with medium expression levels. Investigators can choose analytical procedures according to their available computer resources, or whether genes of high or low expression levels are of interest. If computer resources are abundant, one can utilize multiple procedures to obtain the intersection of results to get the most reliable DEGs, or to obtain a combination of results to get a more comprehensive DE profile for transcriptomes.

## Supplementary Information

**Additional file 1.**

**Additional file 2. **

**Additional file 3. **

**Additional file 4. **

**Additional file 5. **

**Additional file 6. **

**Additional file 7. **

**Additional file 8. **

**Additional file 9. **

## Data Availability

All summary data included in the results is included in supplementary materials, and the datasets analyzed in this study are available from the corresponding authors upon reasonable request. The mouse RNA-seq dataset used in this study can be found in NCBI, GSE111708 [31]. The human, rat and macaque RNA-seq datasets used in this study can be found in ERP001942 [32–34], GSE159668 [35] and GSE184949 [36], respectively.

## Abbreviations

DEGs: Differentially expressed genes
FPKM: Fragments per kilobase of transcript per million mapped reads
DE: Differential expression
GC: Guanine-cytosine
PFC: Prefrontal cortex
RAM: Random access memory
qRT-PCR: Quantitative real-time polymerase chain reaction
VR: Verification rates

## References

[1] Seyednasrollah F, Laiho A, Elo LL (2015). Comparison of software packages for detecting differential expression in RNA-seq studies. Brief Bioinform.

[2] Sahraeian SME, Mohiyuddin M, Sebra R, Tilgner H, Afshar PT, Au KF, Bani Asadi N, Gerstein MB, Wong WH, Snyder MP (2017). Gaining comprehensive biological insight into the transcriptome by performing a broad-spectrum RNA-seq analysis. Nat Commun.

[3] Stark R, Grzelak M, Hadfield J (2019). RNA sequencing: the teenage years. Nat Rev Genet.

[4] Cock PJ, Fields CJ, Goto N, Heuer ML, Rice PM (2010). The Sanger FASTQ file format for sequences with quality scores, and the Solexa/Illumina FASTQ variants. Nucleic Acids Res.

[5] Kim D, Pertea G, Trapnell C, Pimentel H, Kelley R, Salzberg SL (2013). TopHat2 accurate alignment of transcriptomes in the presence of insertions, deletions and gene fusions. Genome Biology.

[6] Kim D, Langmead B, Salzberg SL (2015). HISAT: a fast spliced aligner with low memory requirements. Nat Methods.

[7] Dobin A, Davis CA, Schlesinger F, Drenkow J, Zaleski C, Jha S, Batut P, Chaisson M, Gingeras TR (2013). STAR: ultrafast universal RNA-seq aligner. Bioinformatics.

[8] Pertea M, Kim D, Pertea GM, Leek JT, Salzberg SL (2016). Transcript-level expression analysis of RNA-seq experiments with HISAT. StringTie and Ballgown Nat Protoc.

[9] Trapnell C, Roberts A, Goff L, Pertea G, Kim D, Kelley DR, Pimentel H, Salzberg SL, Rinn JL, Pachter L (2012). Differential gene and transcript expression analysis of RNA-seq experiments with TopHat and Cufflinks. Nat Protoc.

[10] Bray NL, Pimentel H, Melsted P, Pachter L (2016). Near-optimal probabilistic RNA-seq quantification. Nat Biotechnol.

[11] Patro R, Duggal G, Love MI, Irizarry RA, Kingsford C (2017). Salmon provides fast and bias-aware quantification of transcript expression. Nat Methods.

[12] Williams CR, Baccarella A, Parrish JZ, Kim CC (2017). Empirical assessment of analysis workflows for differential expression analysis of human samples using RNA-Seq. BMC Bioinformatics.

[13] Robert C, Watson M (2015). Errors in RNA-Seq quantification affect genes of relevance to human disease. Genome Biol.

[14] Schmid MW, Grossniklaus U (2015). Rcount: simple and flexible RNA-Seq read counting. Bioinformatics.

[15] Anders S, Pyl PT, Huber W (2015). HTSeq–a Python framework to work with high-throughput sequencing data. Bioinformatics.

[16] Bullard JH, Purdom E, Hansen KD, Dudoit S (2010). Evaluation of statistical methods for normalization and differential expression in mRNA-Seq experiments. BMC Bioinformatics.

[17] Dillies MA, Rau A, Aubert J, Hennequet-Antier C, Jeanmougin M, Servant N, Keime C, Marot G, Castel D, Estelle J (2013). A comprehensive evaluation of normalization methods for Illumina high-throughput RNA sequencing data analysis. Brief Bioinform.

[18] Teng M, Love MI, Davis CA, Djebali S, Dobin A, Graveley BR, Li S, Mason CE, Olson S, Pervouchine D (2016). A benchmark for RNA-seq quantification pipelines. Genome Biol.

[19] Li X, Brock GN, Rouchka EC, Cooper NGF, Wu D, O'Toole TE, Gill RS, Eteleeb AM, O'Brien L, Rai SN (2017). A comparison of per sample global scaling and per gene normalization methods for differential expression analysis of RNA-seq data. PLoS One.

[20] Mortazavi A, Williams BA, McCue K, Schaeffer L, Wold B (2008). Mapping and quantifying mammalian transcriptomes by RNA-Seq. Nat Methods.

[21] Risso D, Schwartz K, Sherlock G, Dudoit S (2011). GC-content normalization for RNA-Seq data. BMC Bioinformatics.

[22] Wagner GP, Kin K, Lynch VJ (2012). Measurement of mRNA abundance using RNA-seq data: RPKM measure is inconsistent among samples. Theory Biosci.

[23] Love MI, Huber W, Anders S (2014). Moderated estimation of fold change and dispersion for RNA-seq data with DESeq2. Genome Biol.

[24] Robinson MD, Oshlack A (2010). A scaling normalization method for differential expression analysis of RNA-seq data. Genome Biol.

[25] Law CW, Chen Y, Shi W, Smyth GK (2014). voom: Precision weights unlock linear model analysis tools for RNA-seq read counts. Genome Biol.

[26] Frazee AC, Pertea G, Jaffe AE, Langmead B, Salzberg SL, Leek JT (2015). Ballgown bridges the gap between transcriptome assembly and expression analysis. Nat Biotechnol.

[27] Pimentel H, Bray NL, Puente S, Melsted P, Pachter L (2017). Differential analysis of RNA-seq incorporating quantification uncertainty. Nat Methods.

[28] Nookaew I, Papini M, Pornputtapong N, Scalcinati G, Fagerberg L, Uhlen M, Nielsen J (2012). A comprehensive comparison of RNA-Seq-based transcriptome analysis from reads to differential gene expression and cross-comparison with microarrays: a case study in Saccharomyces cerevisiae. Nucleic Acids Res.

[29] Teng M, Love MI, Davis CA, Djebali S, Dobin A, Graveley BR, Li S, Mason CE, Olson S, Pervouchine D (2016). Erratum to: A benchmark for RNA-seq quantification pipelines. Genome Biol.

[30] Corchete LA, Rojas EA, Alonso-Lopez D, De Las RJ, Gutierrez NC, Burguillo FJ (2020). Systematic comparison and assessment of RNA-seq procedures for gene expression quantitative analysis. Sci Rep.

[31] Zhao J, Liu X, Huo C, Zhao T, Ye H (2018). Abnormalities in Prefrontal Cortical Gene Expression Profiles Relevant to Schizophrenia in MK-801-Exposed C57BL/6 Mice. Neuroscience.

[32] Ferreira PG, Oti M, Barann M, Wieland T, Ezquina S, Friedlander MR, Rivas MA, Esteve-Codina A, Consortium G, Rosenstiel P (2016). Sequence variation between 462 human individuals fine-tunes functional sites of RNA processing. Sci Rep.

[33] Vicente CT, Edwards SL, Hillman KM, Kaufmann S, Mitchell H, Bain L, Glubb DM, Lee JS, French JD, Ferreira MA (2015). Long-Range Modulation of PAG1 Expression by 8q21 Allergy Risk Variants. Am J Hum Genet.

[34] Lappalainen T, Sammeth M, Friedlander MR, Hoen PA, Monlong J, Rivas MA, Gonzalez-Porta M, Kurbatova N, Griebel T, Ferreira PG (2013). Transcriptome and genome sequencing uncovers functional variation in humans. Nature.

[35] Liu J, Deng Y, Fan Z, Xu S, Wei L, Huang X, Xing X, Yang J. Construction and analysis of the abnormal lncRNA-miRNA-mRNA network in hypoxic pulmonary hypertension. Biosci Rep. 2021;41(8). 10.1042/BSR20210021 PMC8390787 34374413

[36] Coleman C, Doyle-Meyers LA, Russell-Lodrigue KE, Golden N, Threeton B, Song K, Pierre G, Baribault C, Bohm RP, Maness NJ (2021). Similarities and Differences in the Acute-Phase Response to SARS-CoV-2 in Rhesus Macaques and African Green Monkeys. Front Immunol.

