# Supplementary material for: A comparison of transcriptome analysis methods with reference genome
Source: BMC Genomics. 2022 Mar 25;23:232. doi: 10.1186/s12864-022-08465-0 (PMC8957167; doi:10.1186/s12864-022-08465-0)
Supplement: Supplementary file 1 — Additional file 1. [file 12864_2022_8465_MOESM1_ESM.pdf]

# Supplementary Figure 1

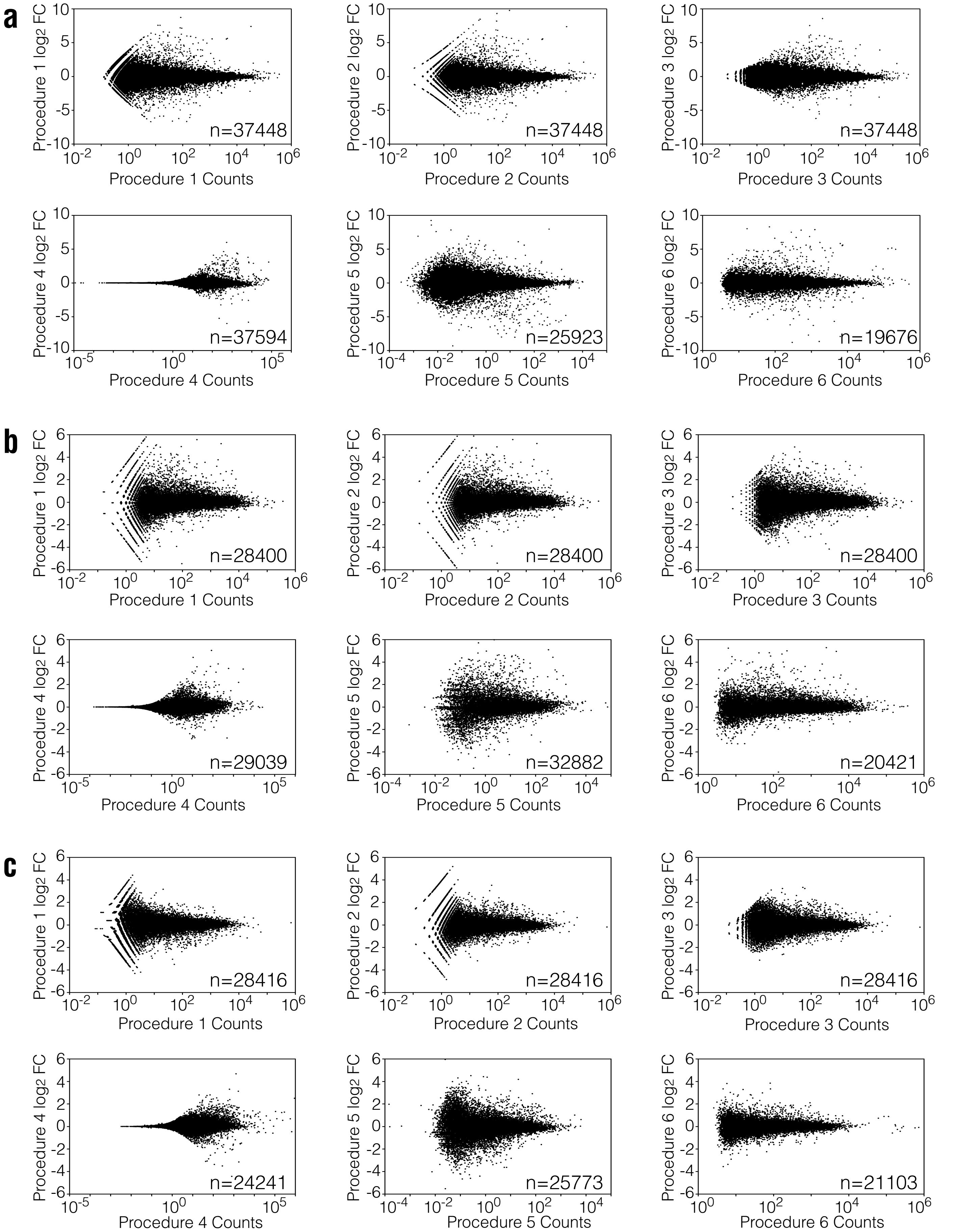


**Supplementary Fig. 1** MA plot of different analysis procedures for (a) human, (b) rat, and (c) macaque datasets, respectively.

# Supplementary Figure 2

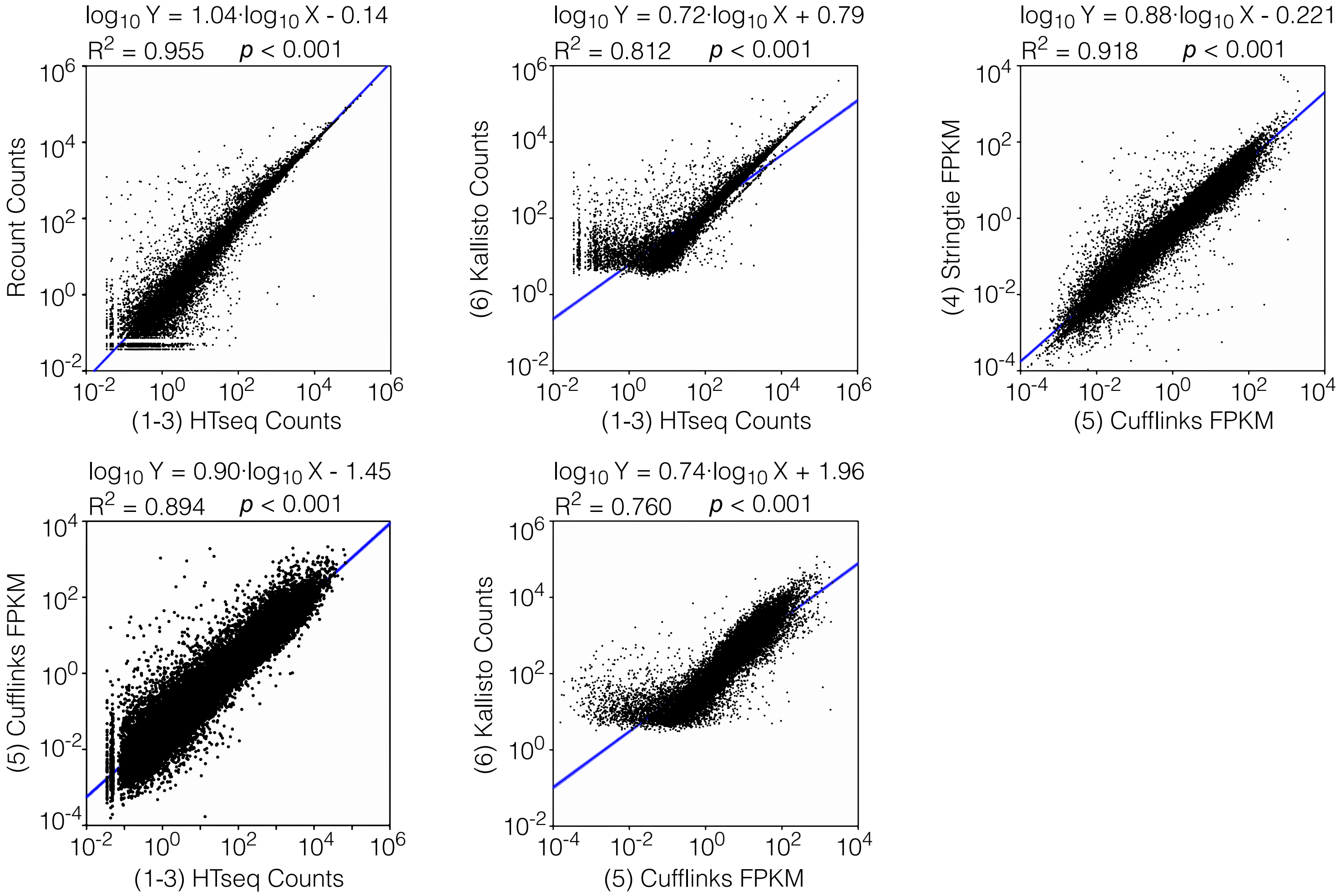


**Supplementary Fig. 2** Comparison of the gene expression values of different algorithms after a logarithmic change in the mouse dataset. The number in brackets represents the procedure number. $R^2$ and *p* was calculated via Pearson's correlation analysis.

# Supplementary Figure 3

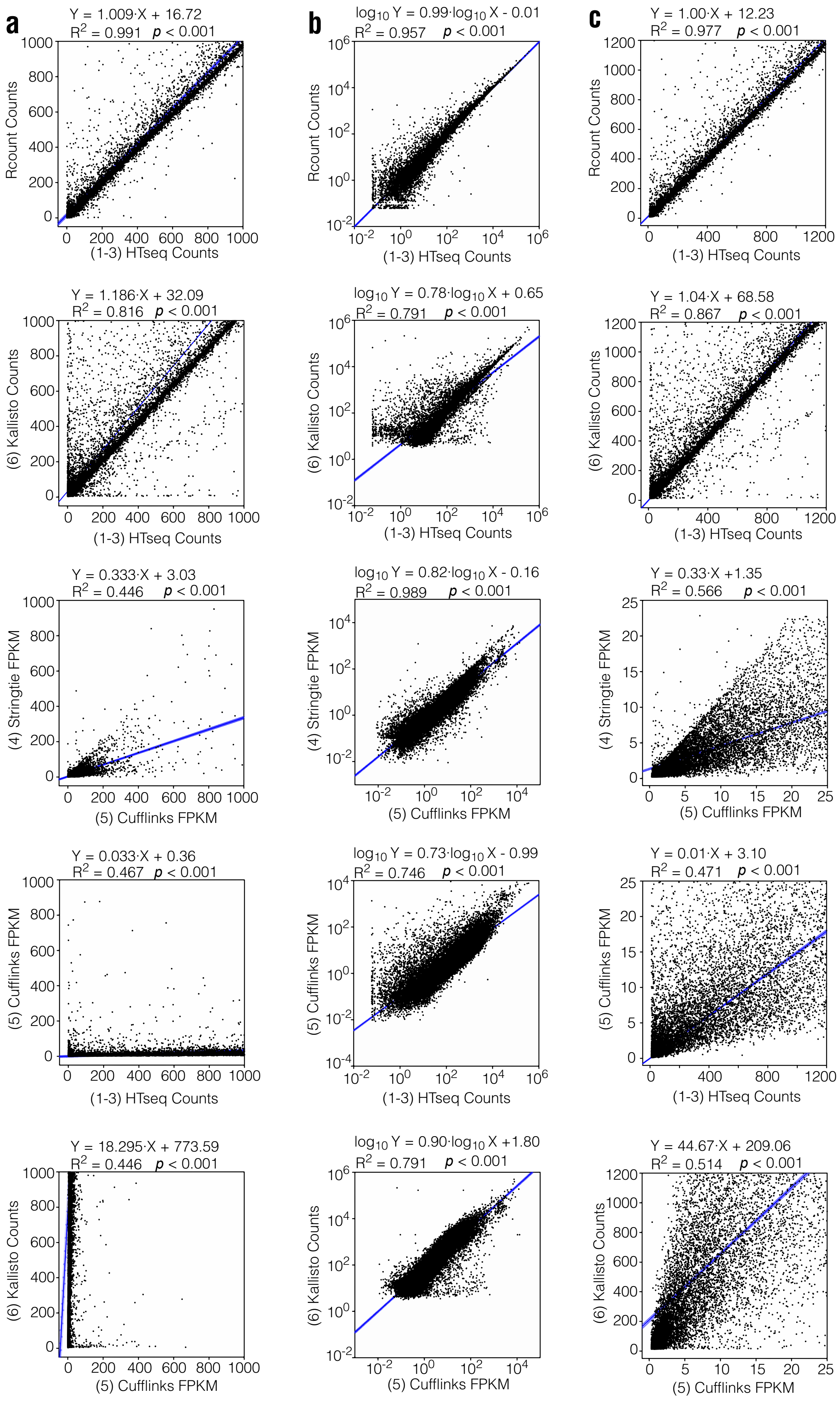


**Supplementary Fig. 3** Comparison of gene expression levels in the human dataset in different conditions. (a) Comparison of gene expression levels evaluated by different quantitative software without any screening. (b) Comparison of the expression values of different algorithms after a logarithmic change. (c) Comparison of gene expression levels obtained with different quantitative software after removing the genes with the upper and lower 10% expression levels. The number in brackets represents the procedure number. $R^2$ and $p$ was calculated via Pearson's correlation analysis.

# Supplementary Figure 4

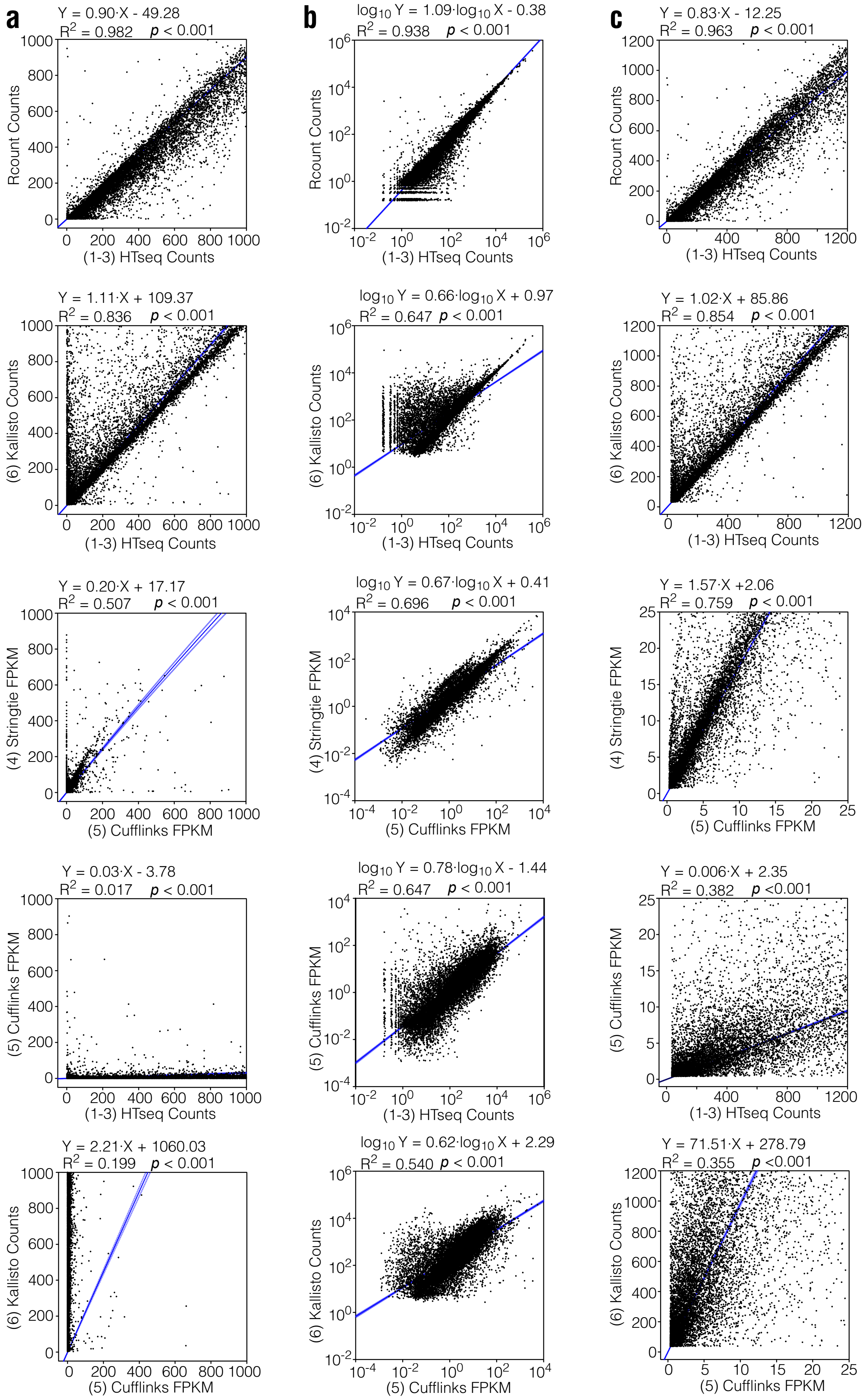


**Supplementary Fig. 4** Comparison of gene expression levels in the rat dataset in different conditions. (a) Comparison of gene expression levels evaluated by different quantitative software without any screening. (b) Comparison of the expression values of different algorithms after a logarithmic change. (c) Comparison of gene expression levels obtained with different quantitative software after removing the genes with the upper and lower 10% expression levels. The number in brackets represents the procedure number. $R^2$ and $p$ was calculated via Pearson's correlation analysis.

# Supplementary Figure 5

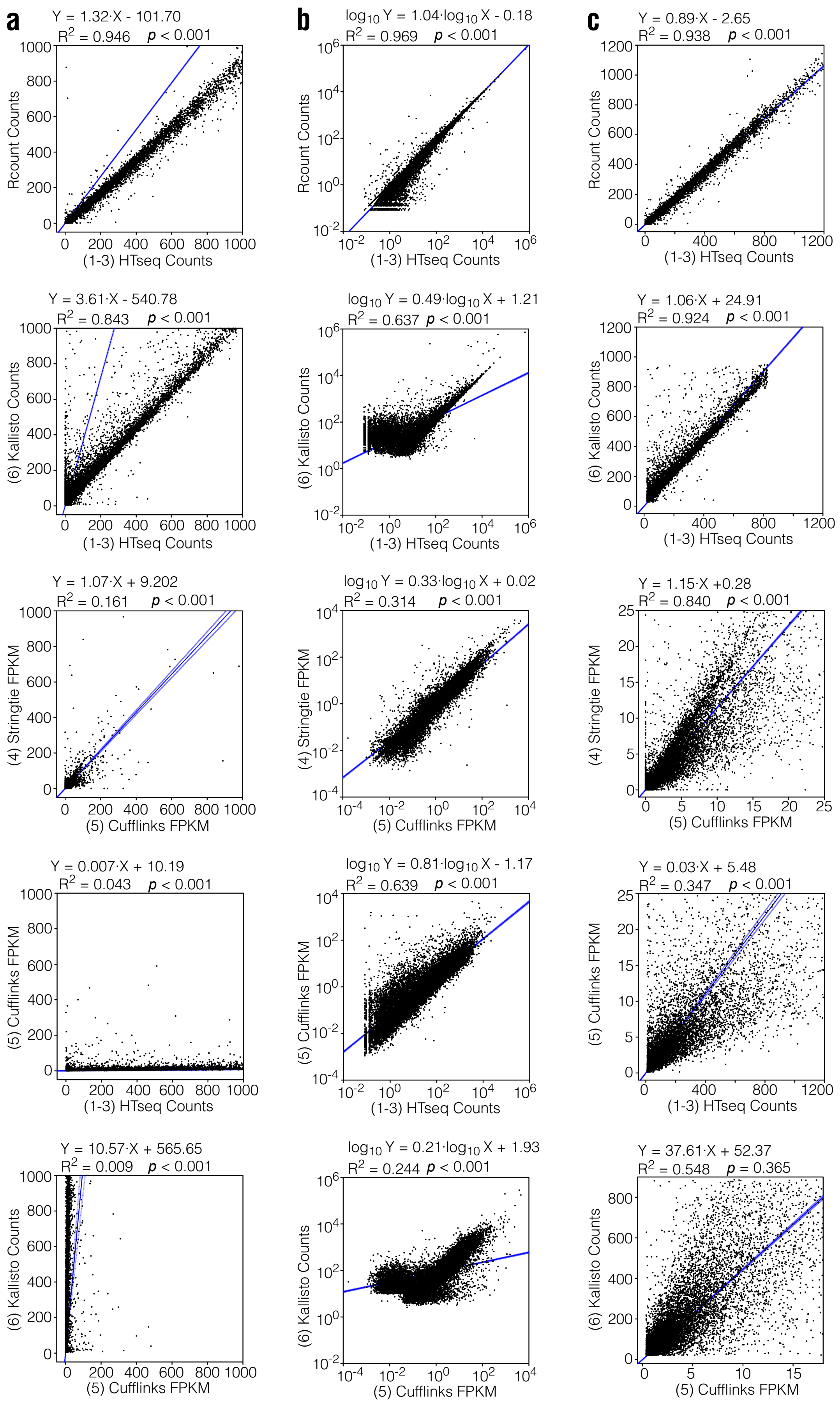


**Supplementary Fig. 5** Comparison of gene expression levels in the macaque dataset in different conditions. (a) Comparison of gene expression levels evaluated by different quantitative software without any screening. (b) Comparison of the expression values of different algorithms after a logarithmic change. (c) Comparison of gene expression levels obtained with different quantitative software after removing the genes with the upper and lower 10% expression levels. The number in brackets represents the procedure number. $R^2$ and $p$ was calculated via Pearson's correlation analysis.
