# Supplementary material for: A comparison of transcriptome analysis methods with reference genome
Source: BMC Genomics. 2022 Mar 25;23:232. doi: 10.1186/s12864-022-08465-0 (PMC8957167; doi:10.1186/s12864-022-08465-0)
Supplement: Supplementary file 2 — Additional file 2. [file 12864_2022_8465_MOESM2_ESM.pdf]

Supplementary Figure 6

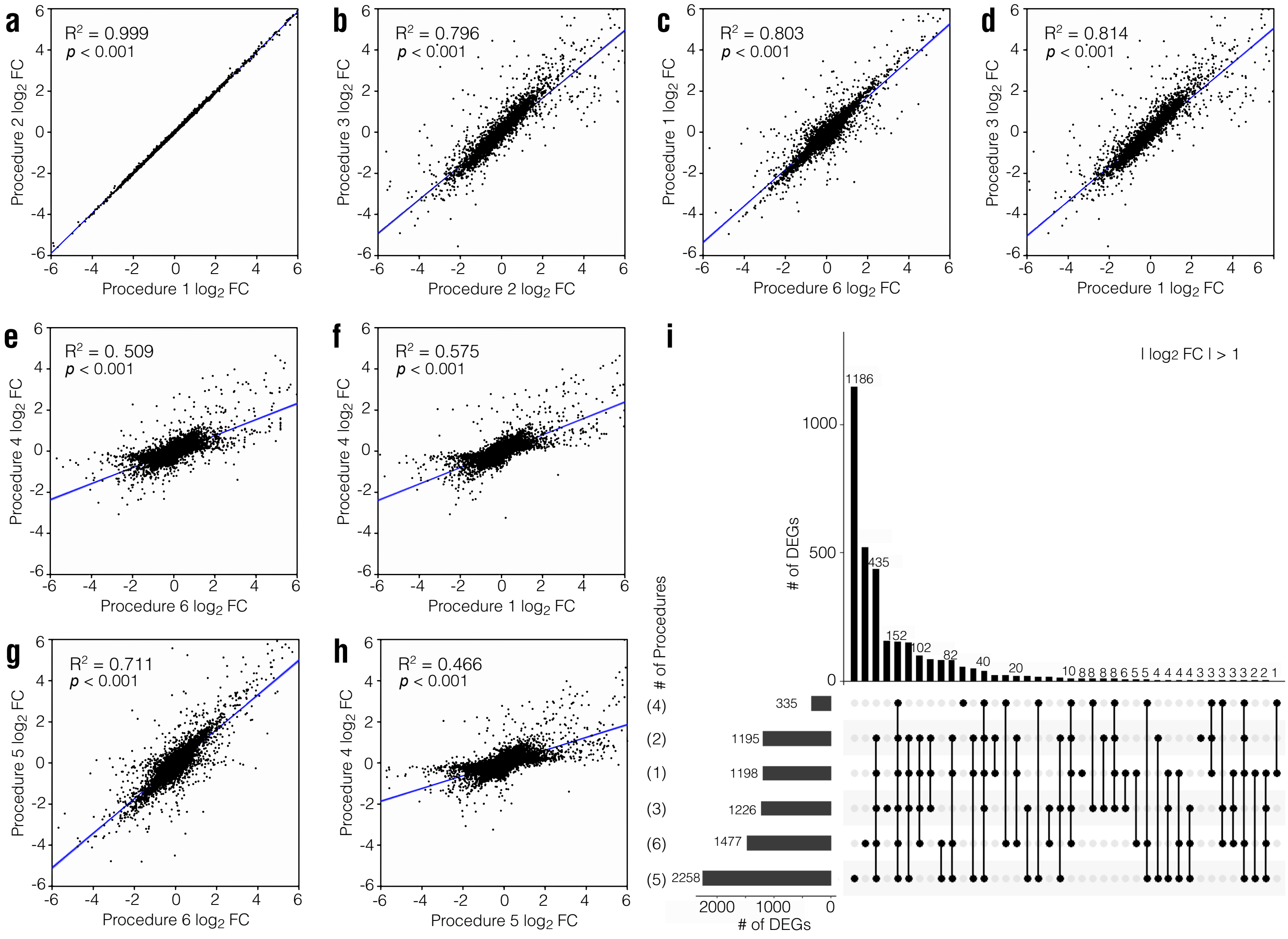

Supplementary Fig. 6 Evaluation and comparison of the fold change of gene expression level obtained from different analysis procedures for the human dataset. (a-h) Comparison of log_2FC obtained from different procedures. (i) Set visualization graphics of DEGs estimated only from the FC among the six procedures. The numbers in brackets represent the procedure number. R^2 and p was calculated via Pearson's correlation analysis.

Supplementary Figure 7

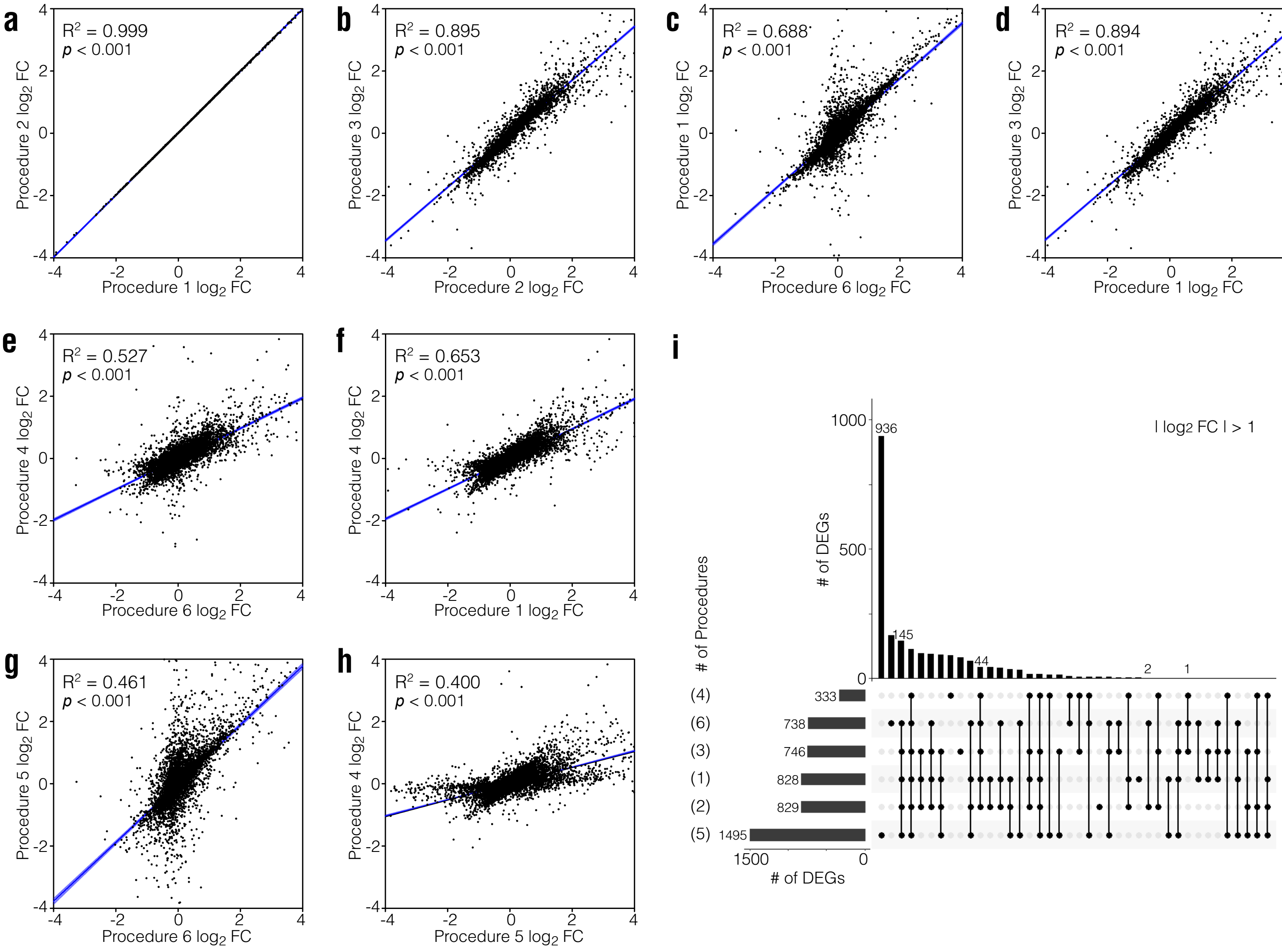

Supplementary Fig. 7 Evaluation and comparison of the fold change of gene expression level obtained from different analysis procedures for the rat dataset. (a-h) Comparison of log_2FC obtained from different procedures. (i) Set visualization graphics of DEGs estimated only from the FC among the six procedures. The numbers in brackets represent the procedure number. R^2 and p was calculated via Pearson's correlation analysis.

Supplementary Figure 8

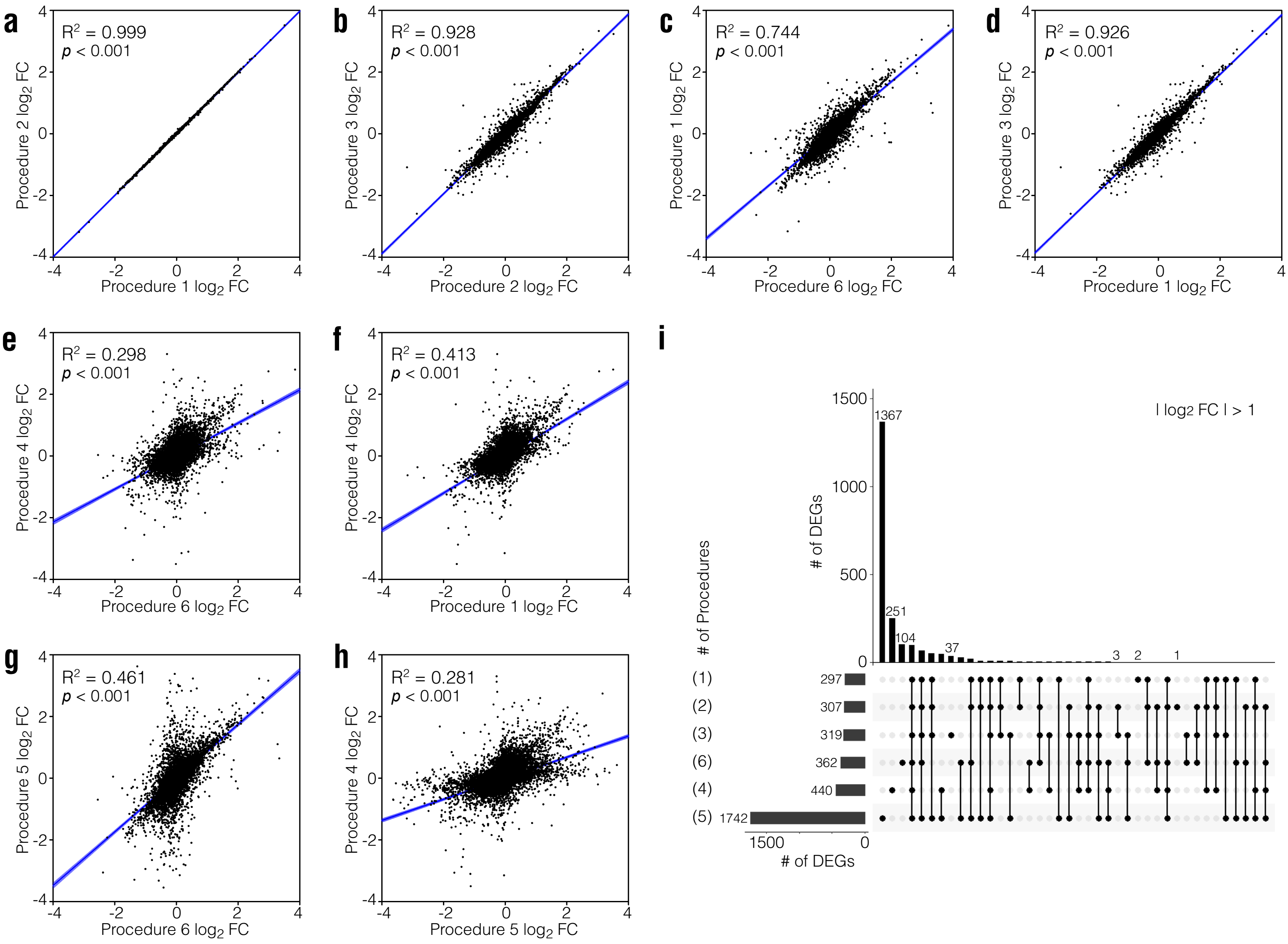

Supplementary Fig. 8 Evaluation and comparison of the fold change of gene expression level obtained from different analysis procedures for the macaque dataset. (a-h) Comparison of log_2FC obtained from different procedures. (i) Set visualization graphics of DEGs estimated only from the FC among the six procedures. The numbers in brackets represent the procedure number. R^2 and p was calculated via Pearson's correlation analysis.
