# Supplementary material for: A comparison of transcriptome analysis methods with reference genome
Source: BMC Genomics. 2022 Mar 25;23:232. doi: 10.1186/s12864-022-08465-0 (PMC8957167; doi:10.1186/s12864-022-08465-0)
Supplement: Supplementary file 3 — Additional file 3. [file 12864_2022_8465_MOESM3_ESM.pdf]

Supplementary Figure 9

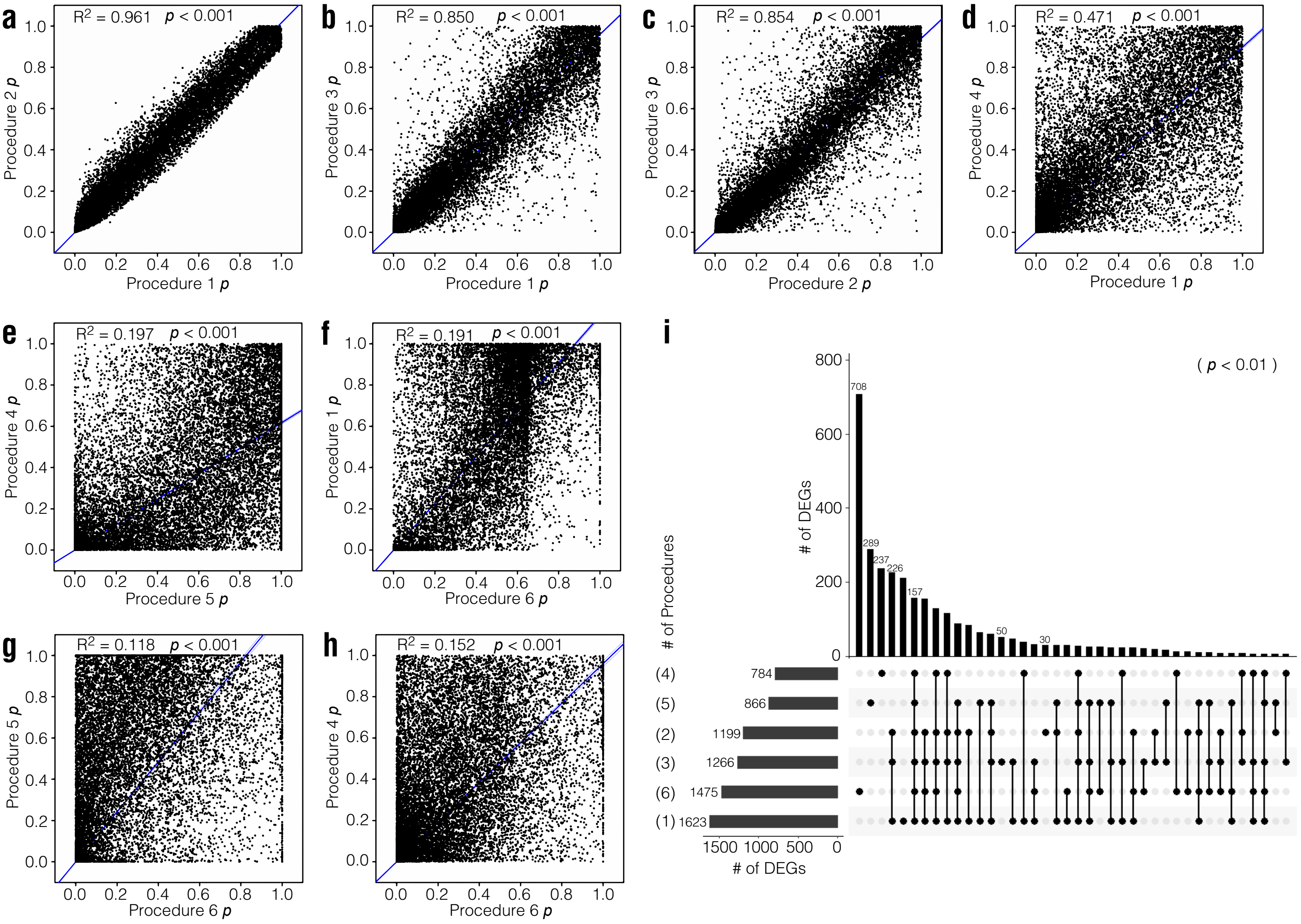

Supplementary Fig. 9 Evaluation and comparison of *p* values for different analysis procedure for the human data-set. (a-h) Comparison of *p* values obtained from different procedures. R^2 and *p* was calculated via Pearson's correlation analysis. (i) Set visualization graphics of DEGs from six procedures when $p<0.01$ was used as the threshold to define DEGs. The numbers in brackets represent the procedure number.

Supplementary Figure 10

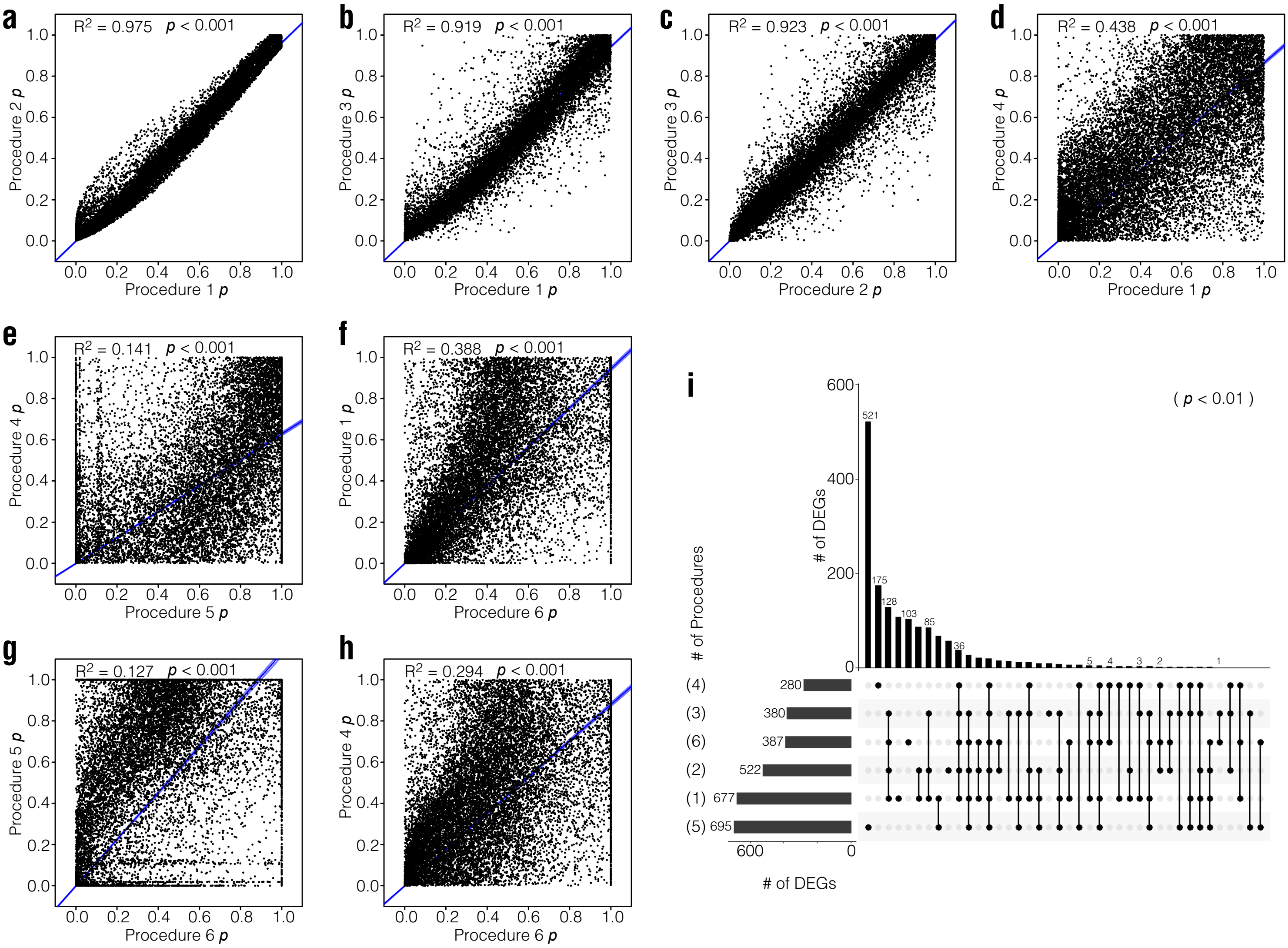

Supplementary Fig. 10 Evaluation and comparison of *p* values for different analysis procedure for the rat dataset. (a-h) Comparison of *p* values obtained from different procedures. R^2 and *p* was calculated via Pearson's correlation analysis. (i) Set visualization graphics of DEGs from six procedures when p<0.01 was used as the threshold to define DEGs. The numbers in brackets represent the procedure number.

Supplementary Figure 11

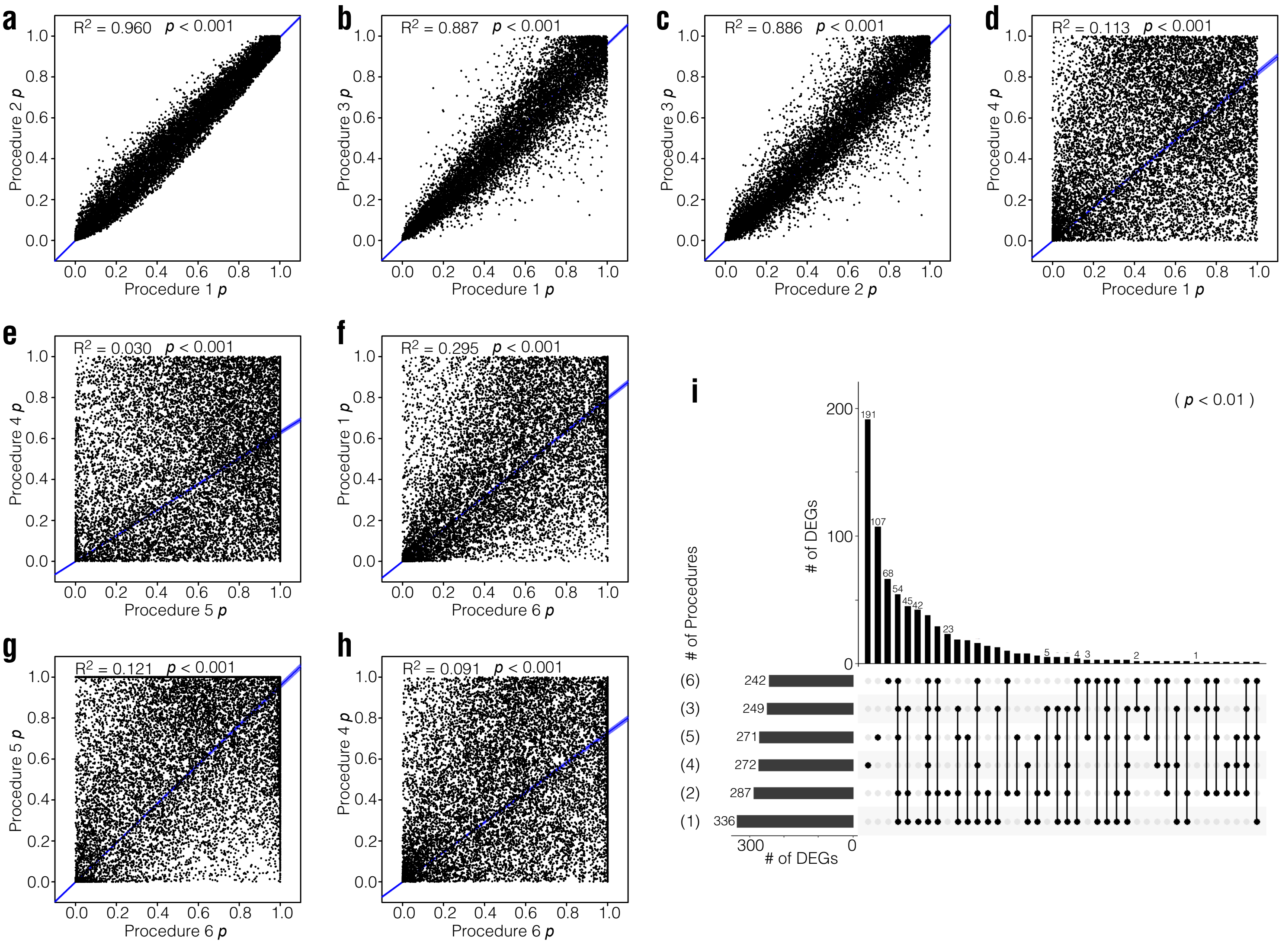

Supplementary Fig. 11 Evaluation and comparison of *p* values for different analysis procedure for the macaque dataset. (a-h) Comparison of *p* values obtained from different procedures. R^2 and *p* was calculated via Pearson's correlation analysis. (i) Set visualization graphics of DEGs from six procedures when $p<0.01$ was used as the threshold to define DEGs. The numbers in brackets represent the procedure number.
