# Supplementary material for: A comparison of transcriptome analysis methods with reference genome
Source: BMC Genomics. 2022 Mar 25;23:232. doi: 10.1186/s12864-022-08465-0 (PMC8957167; doi:10.1186/s12864-022-08465-0)
Supplement: Supplementary file 4 — Additional file 4. [file 12864_2022_8465_MOESM4_ESM.pdf]

Supplementary Figure 12

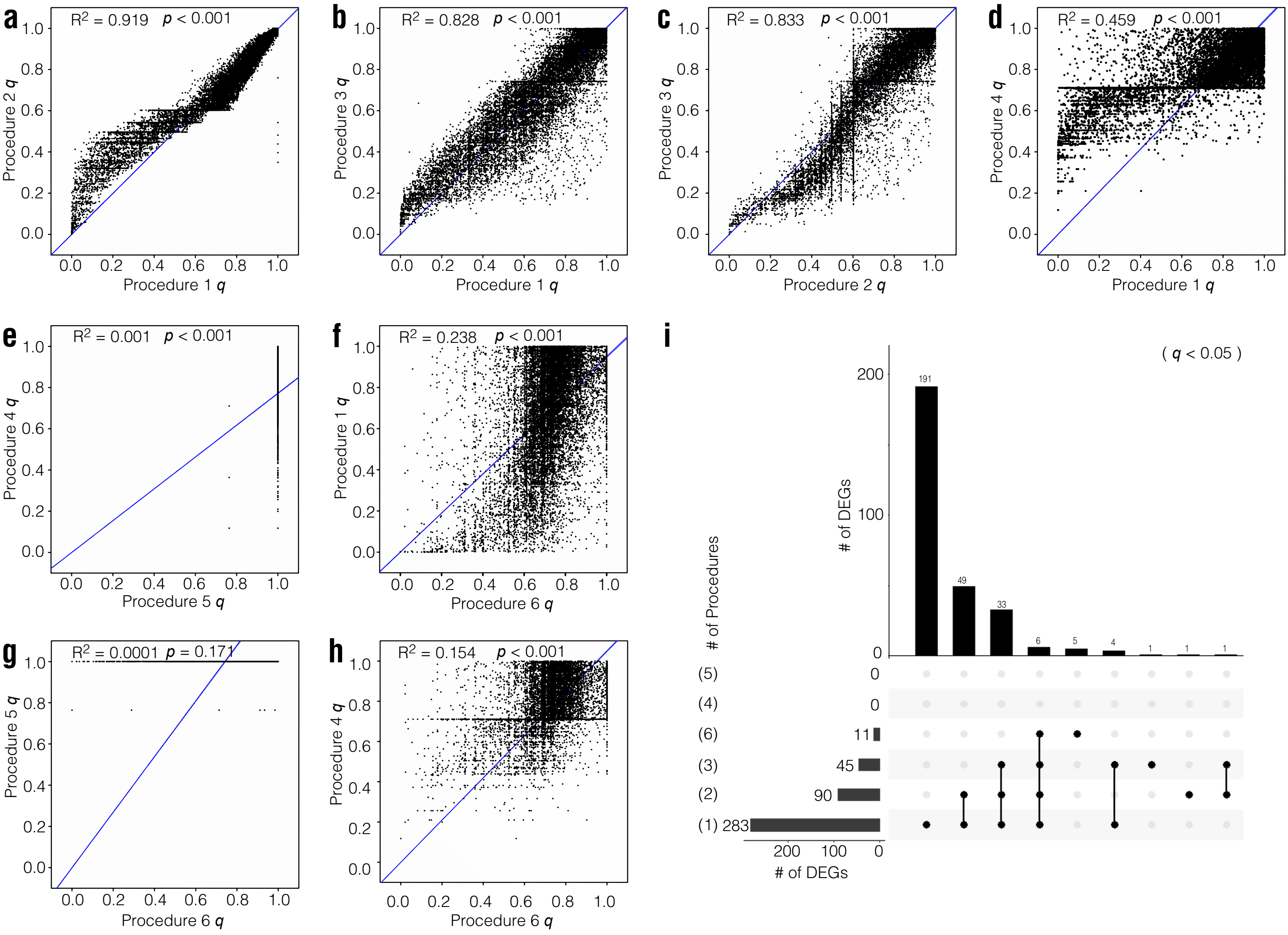

Supplementary Fig. 12 Evaluation and comparison of *q* values for different analysis procedure for the mouse dataset. (a-h) Comparison of *q* values obtained from different procedures. R^2 and *p* was calculated via Pearson's correlation analysis. (i) Set visualization graphics of DEGs from six procedures when $q<0.05$ was used as the threshold to define DEGs. The numbers in brackets represent the procedure number.

Supplementary Figure 13

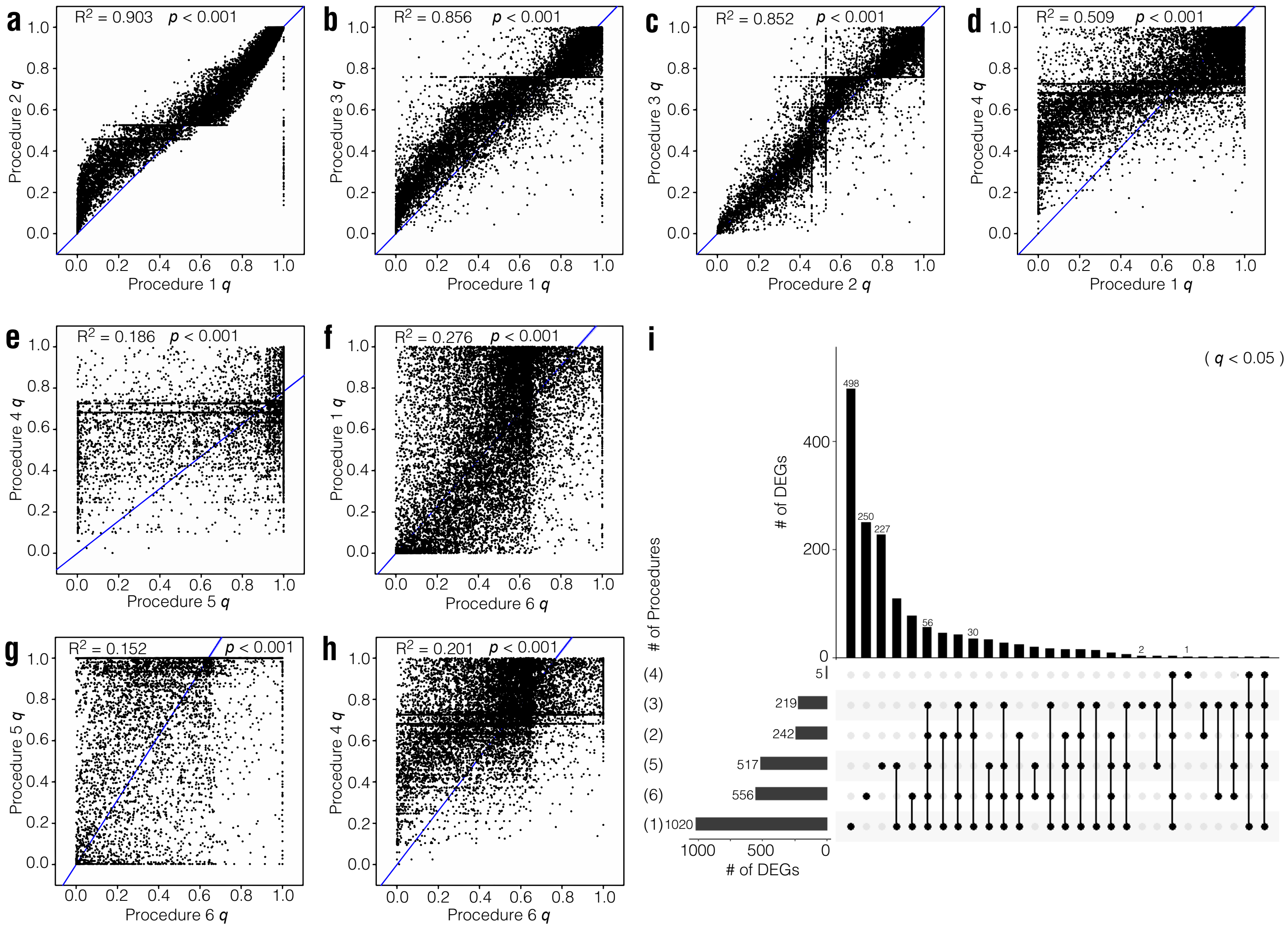

Supplementary Fig. 13 Evaluation and comparison of *q* values for different analysis procedure for the human data-set. (a-h) Comparison of *q* values obtained from different procedures. R^2 and *p* was calculated via Pearson's correlation analysis. (i) Set visualization graphics of DEGs from six procedures when q<0.05 was used as the threshold to define DEGs. The numbers in brackets represent the procedure number.

Supplementary Figure 14

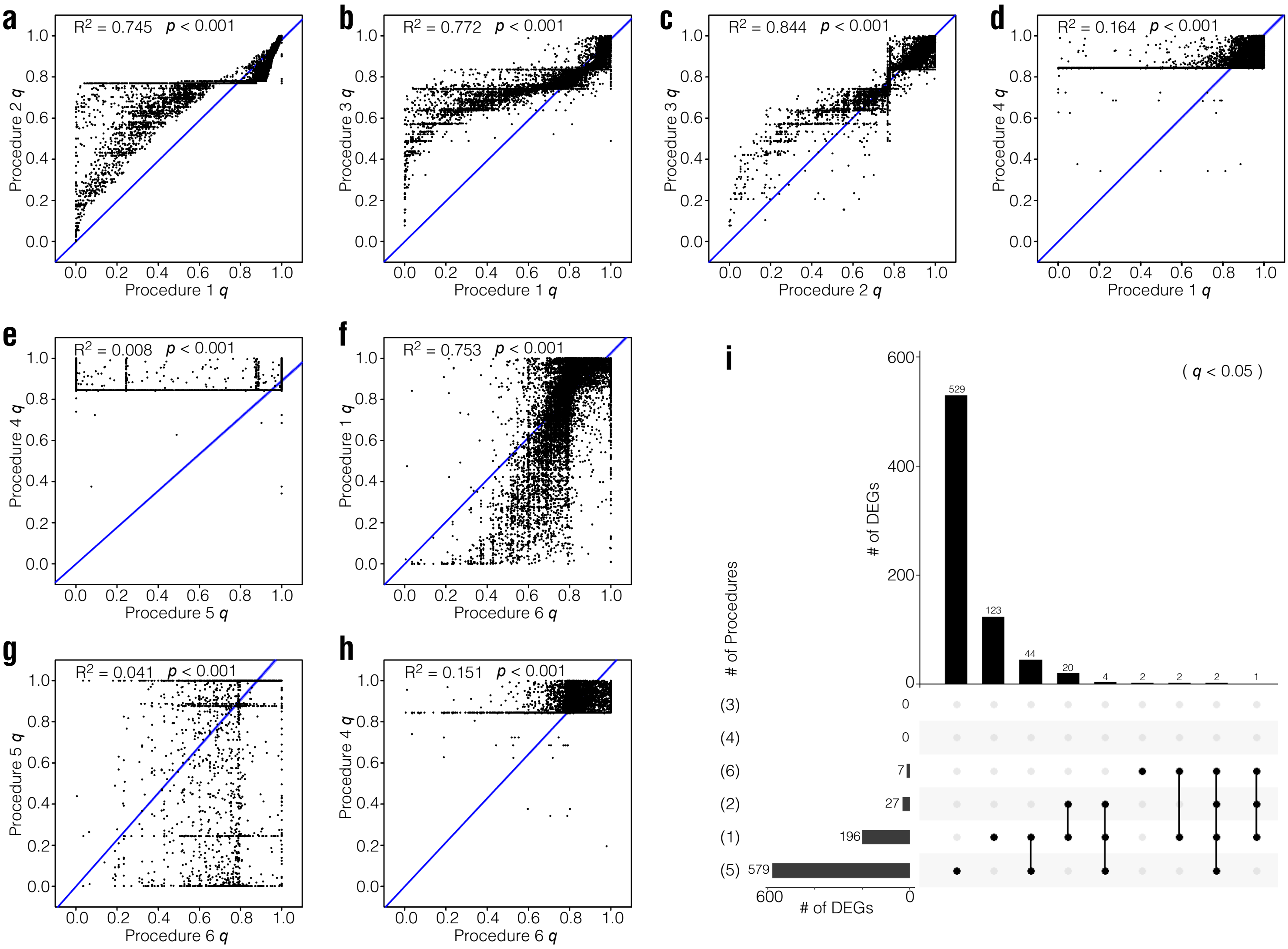

Supplementary Fig. 14 Evaluation and comparison of *q* values for different analysis procedure for the rat dataset. (a-h) Comparison of *q* values obtained from different procedures. R^2 and *p* was calculated via Pearson's correlation analysis. (i) Set visualization graphics of DEGs from six procedures when $q<0.05$ was used as the threshold to define DEGs. The numbers in brackets represent the procedure number.

Supplementary Figure 15

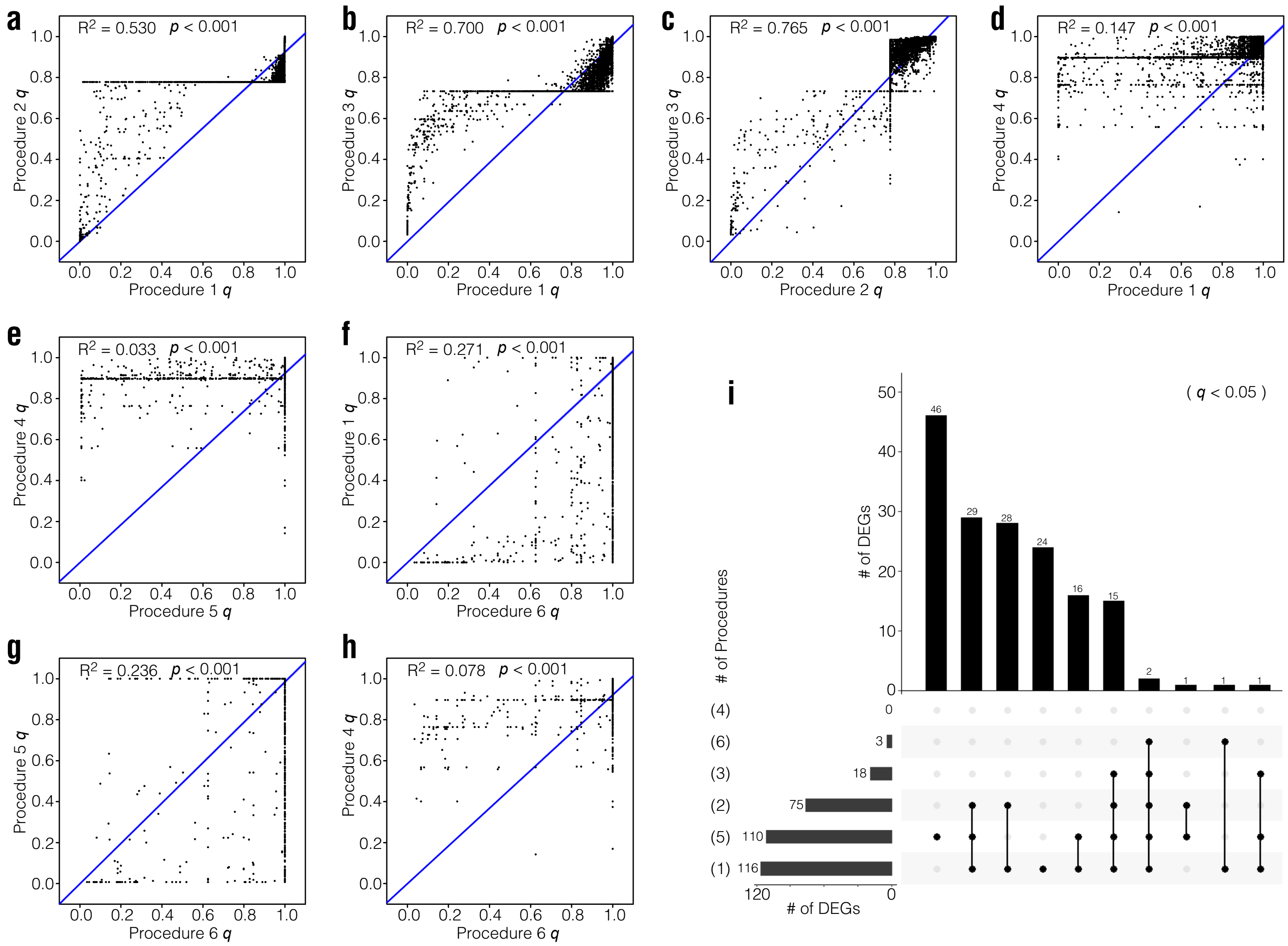

Supplementary Fig. 15 Evaluation and comparison of *q* values for different analysis procedure for the macaque dataset. (a-h) Comparison of *q* values obtained from different procedures. R^2 and *p* was calculated via Pearson's correlation analysis. (i) Set visualization graphics of DEGs from six procedures when q<0.05 was used as the threshold to define DEGs. The numbers in brackets represent the procedure number.
