# Supplementary material for: A comparison of transcriptome analysis methods with reference genome
Source: BMC Genomics. 2022 Mar 25;23:232. doi: 10.1186/s12864-022-08465-0 (PMC8957167; doi:10.1186/s12864-022-08465-0)
Supplement: Supplementary file 5 — Additional file 5. [file 12864_2022_8465_MOESM5_ESM.pdf]

Supplementary Figure 16

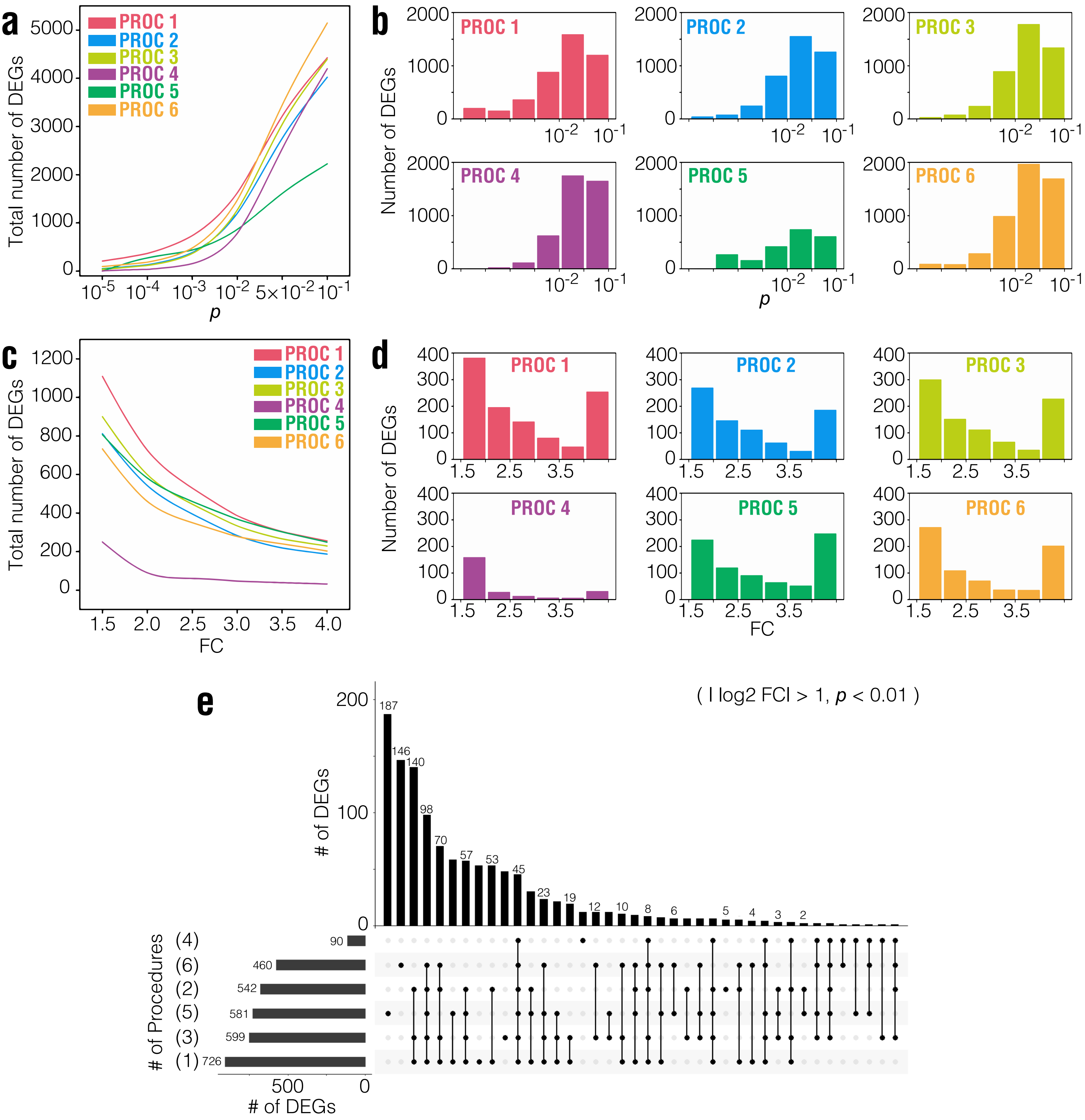

Supplementary Fig. 16 Number of DEGs with combination of FC and *p*value for the human dataset. (a) The line chart reflects the total number of DEGs estimated by different procedures with $|Log_2FC|>1$ and different *p* value. (b) The histogram reflects the interval number of DEGs estimated by different procedures with $|Log_2FC|>1$ and different *p* value. (c) The line chart reflects the total number of DEGs estimated by different procedures with differences procedures with $p<0.01$ and different $|Log_2FC|$. (d) The histogram reflects the interval number of DEGs estimated by different procedures with differences procedures with $p<0.01$ and different $|Log_2FC|$. (e) Set visualization graphics of DEGs when $|Log_2FC|>1$ and $p<0.01$. The numbers in brackets represent the procedure number.

Supplementary Figure 17

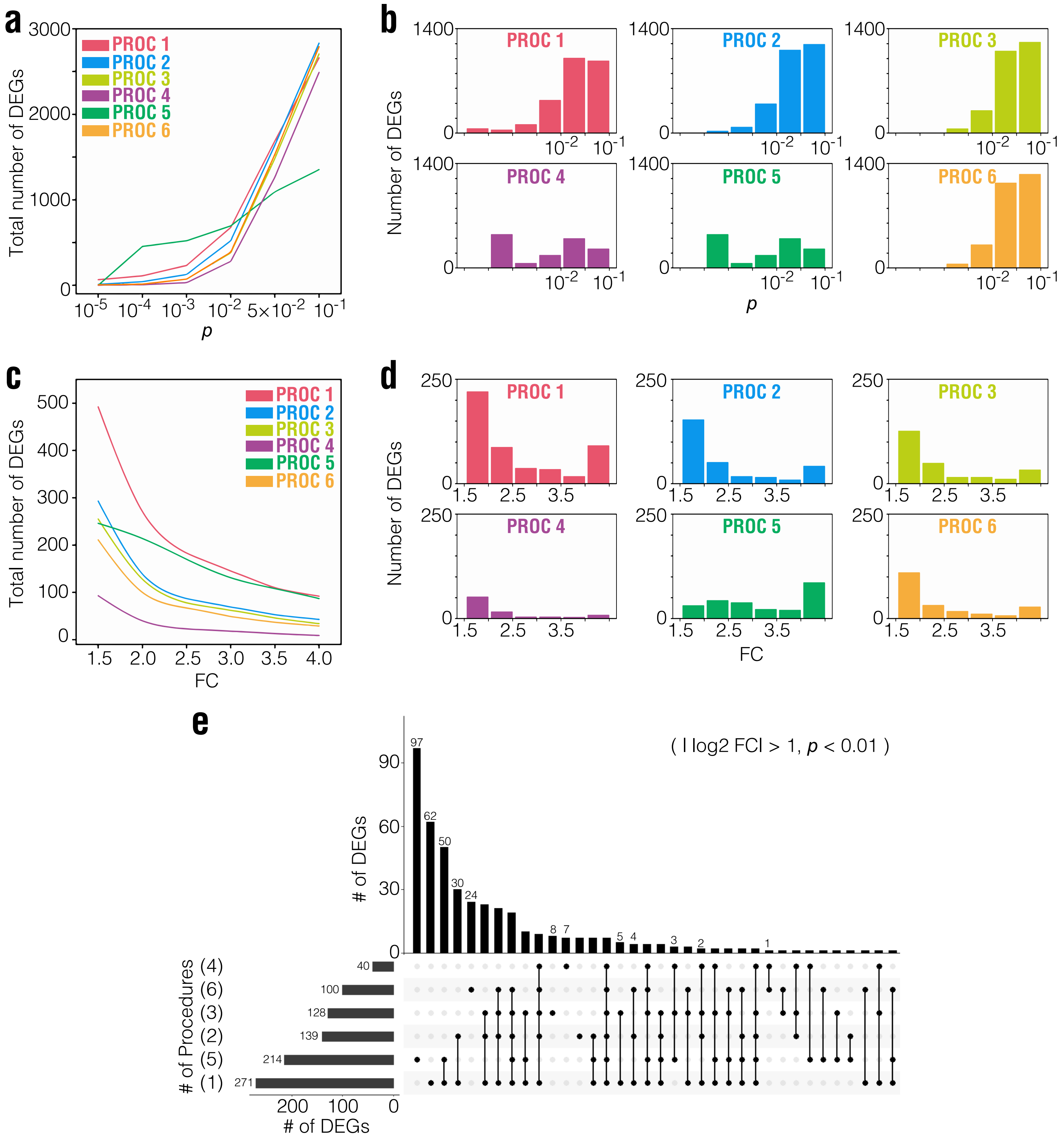

Supplementary Fig. 17 Number of DEGs with combination of FC and *p*value for the rat dataset. (a) The line chart reflects the total number of DEGs estimated by different procedures with $|Log_2FC|>1$ and different *p* value. (b) The histogram reflects the interval number of DEGs estimated by different procedures with $|Log_2FC|>1$ and different *p* value. (c) The line chart reflects the total number of DEGs estimated by different procedures with differences procedures with $p<0.01$ and different $|Log_2FC|$. (d) The histogram reflects the interval number of DEGs estimated by different procedures with differences procedures with $p<0.01$ and different $|Log_2FC|$. (e) Set visualization graphics of DEGs when $|Log_2FC|>1$ and $p<0.01$. The numbers in brackets represent the procedure number.

Supplementary Figure 18

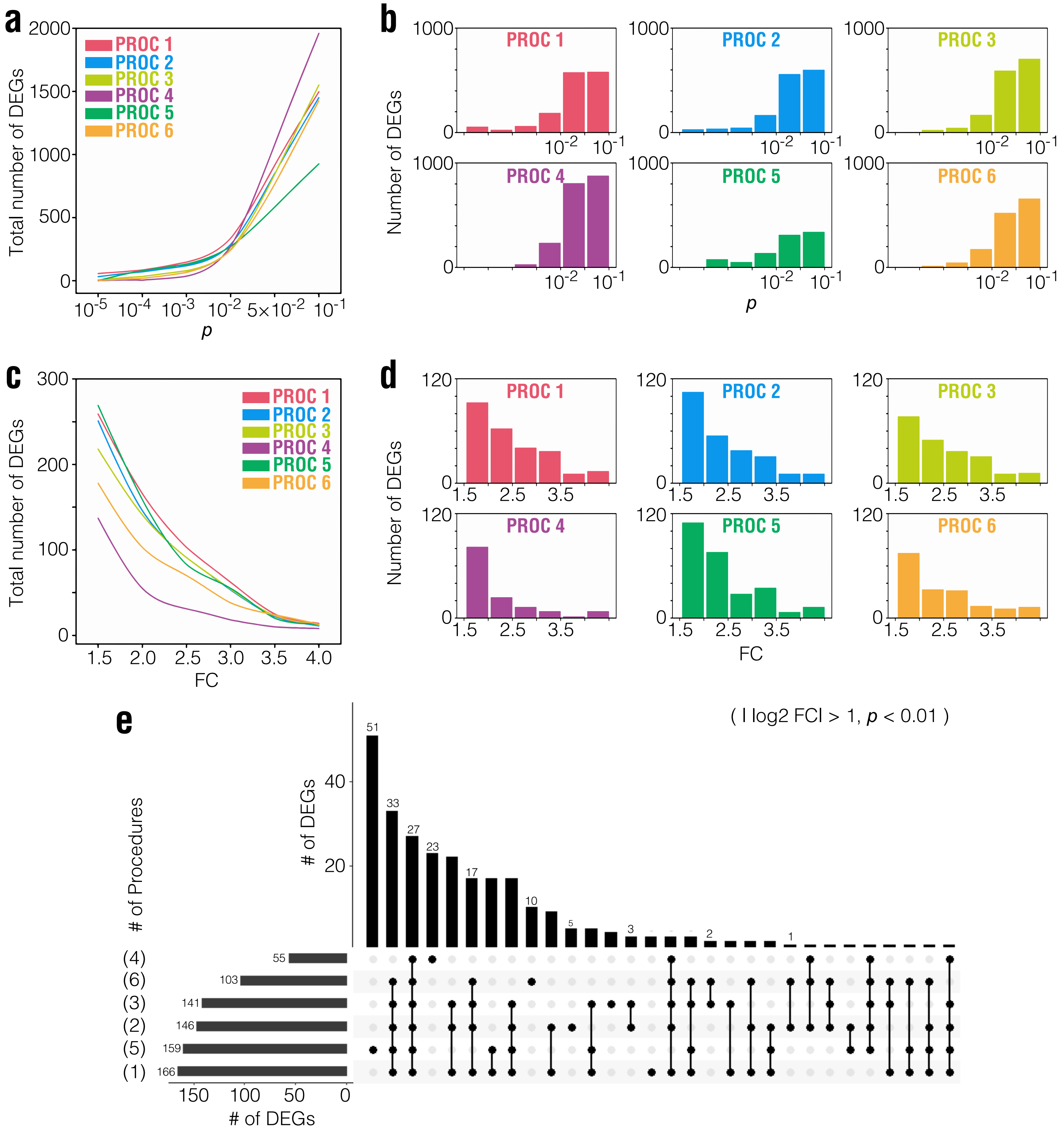

Supplementary Fig. 18 Number of DEGs with combination of FC and *p*value for the macaque dataset. (a) The line chart reflects the total number of DEGs estimated by different procedures with $|Log_2FC|>1$ and different *p* value. (b) The histogram reflects the interval number of DEGs estimated by different procedures with $|Log_2FC|>1$ and different *p* value. (c) The line chart reflects the total number of DEGs estimated by different procedures with differences procedures with $p<0.01$ and different $|Log_2FC|$. (d) The histogram reflects the interval number of DEGs estimated by different procedures with differences procedures with $p<0.01$ and different $|Log_2FC|$. (e) Set visualization graphics of DEGs when $|Log_2FC|>1$ and $p<0.01$. The numbers in brackets represent the procedure number.
