# Supplementary material for: A comparison of transcriptome analysis methods with reference genome
Source: BMC Genomics. 2022 Mar 25;23:232. doi: 10.1186/s12864-022-08465-0 (PMC8957167; doi:10.1186/s12864-022-08465-0)
Supplement: Supplementary file 6 — Additional file 6. [file 12864_2022_8465_MOESM6_ESM.pdf]

Supplementary Figure 19

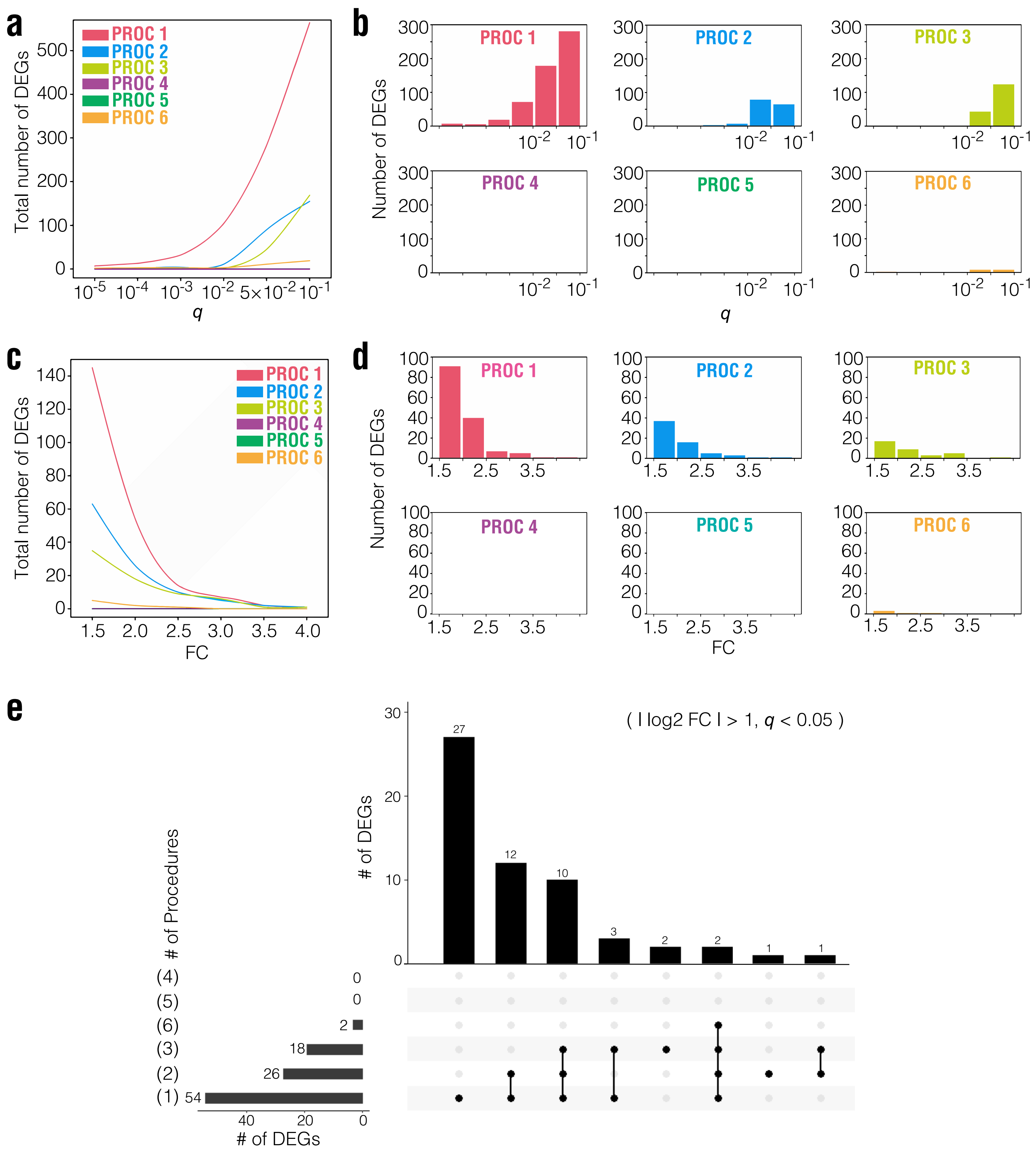

Supplementary Fig. 19 Number of DEGs with combination of FC and q value for the mouse dataset. (a) The line chart reflects the total number of DEGs estimated by different procedures with $|Log_2FC|>1$ and different q value. (b) The histogram reflects the interval number of DEGs estimated by different procedures with $|Log_2FC|>1$ and different q value. (c) The line chart reflects the total number of DEGs estimated by different procedures with differences procedures with $q<0.05$ and different $|Log_2FC|$. (d) The histogram reflects the interval number of DEGs estimated by different procedures with differences procedures with $q<0.05$ and different $|Log_2FC|$. (e) Set visualization graphics of DEGs when $|Log_2FC|>1$ and $q<0.05$. The numbers in brackets represent the procedure number.

Supplementary Figure 20

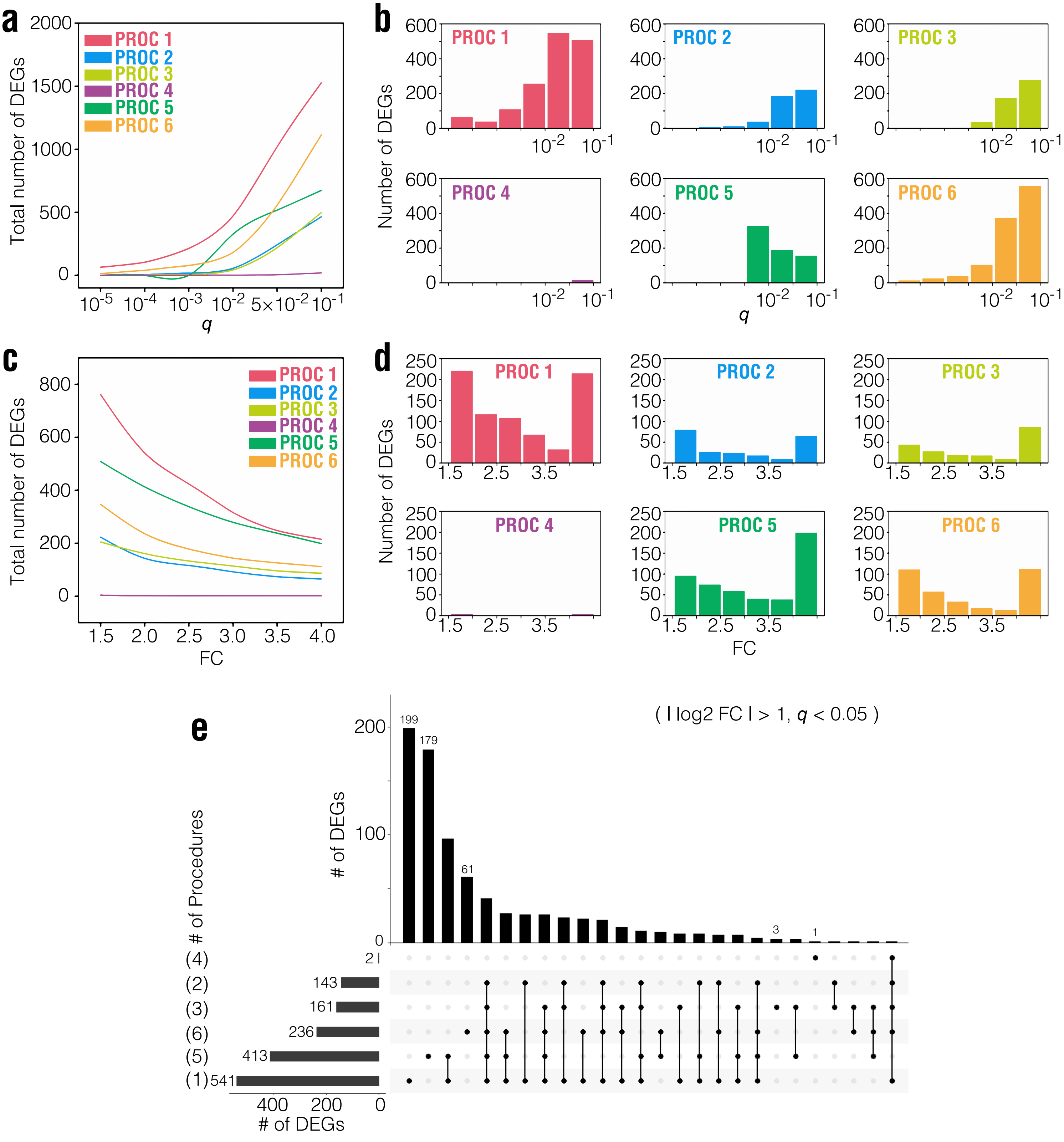

Supplementary Fig. 20 Number of DEGs with combination of FC and q value for the human dataset. (a) The line chart reflects the total number of DEGs estimated by different procedures with $|Log_2FC|>1$ and different q value. (b) The histogram reflects the interval number of DEGs estimated by different procedures with $|Log_2FC|>1$ and different q value. (c) The line chart reflects the total number of DEGs estimated by different procedures with differences procedures with $q<0.05$ and different $|Log_2FC|$. (d) The histogram reflects the interval number of DEGs estimated by different procedures with differences procedures with $q<0.05$ and different $|Log_2FC|$. (e) Set visualization graphics of DEGs when $|Log_2FC|>1$ and $q<0.05$. The numbers in brackets represent the procedure number.

Supplementary Figure 21

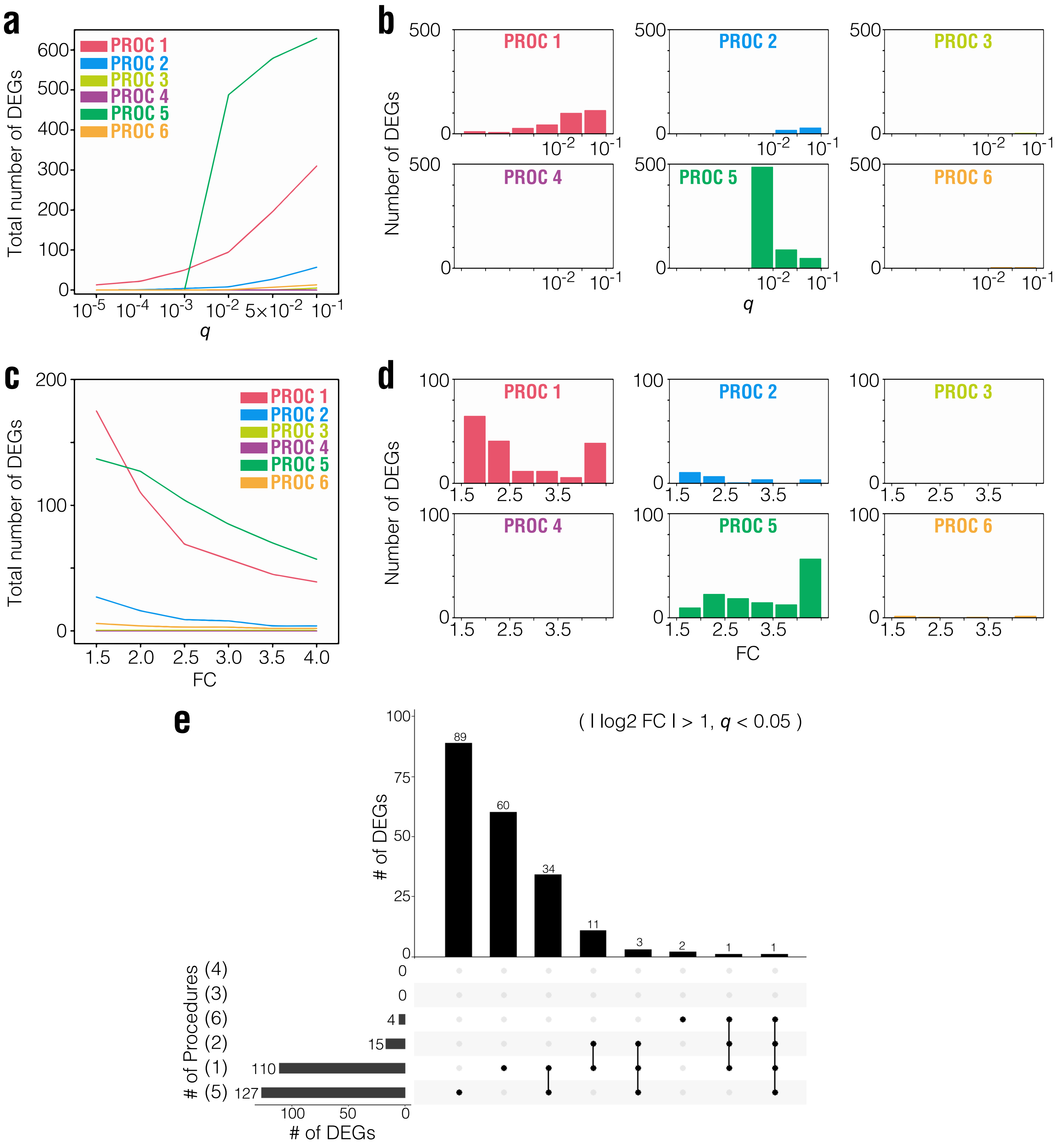

Supplementary Fig. 21 Number of DEGs with combination of FC and q value for the rat dataset. (a) The line chart reflects the total number of DEGs estimated by different procedures with $|Log_2FC|>1$ and different q value. (b) The histogram reflects the interval number of DEGs estimated by different procedures with $|Log_2FC|>1$ and different q value. (c) The line chart reflects the total number of DEGs estimated by different procedures with differences procedures with $q<0.05$ and different $|Log_2FC|$. (d) The histogram reflects the interval number of DEGs estimated by different procedures with differences procedures with $q<0.05$ and different $|Log_2FC|$. (e) Set visualization graphics of DEGs when $|Log_2FC| > 1$ and $q<0.05$. The numbers in brackets represent the procedure number.

Supplementary Figure 22

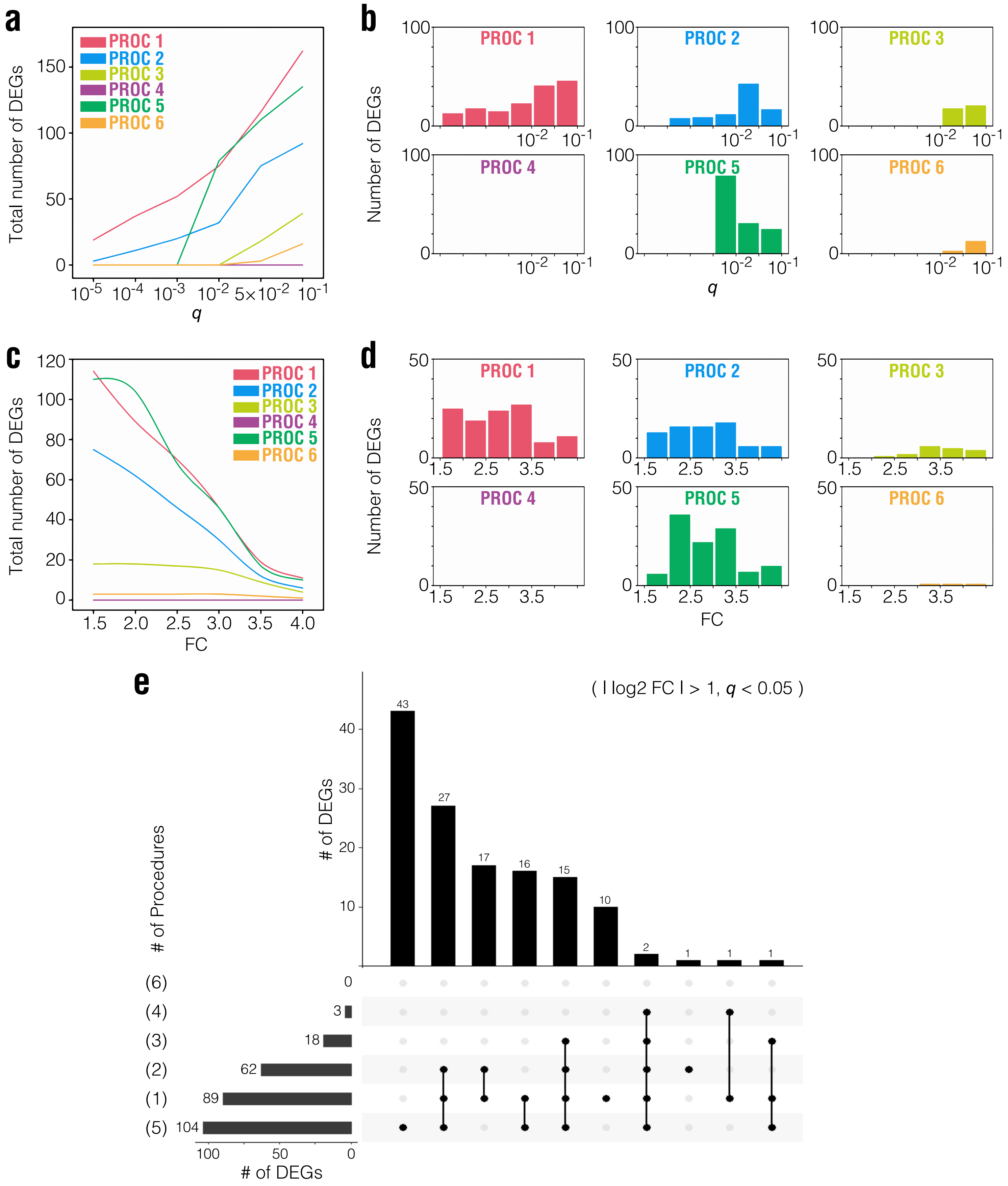

Supplementary Fig. 22 Number of DEGs with combination of FC and *q* value for the macaque dataset. (a) The line chart reflects the total number of DEGs estimated by different procedures with $|Log_2FC|>1$ and different *q* value. (b) The histogram reflects the interval number of DEGs estimated by different procedures with $|Log_2FC|>1$ and different *q* value. (c) The line chart reflects the total number of DEGs estimated by different procedures with differences procedures with $q<0.05$ and different $|Log_2FC|$. (d) The histogram reflects the interval number of DEGs estimated by different procedures with differences procedures with $q<0.05$ and different $|Log_2FC|$. (e) Set visualization graphics of DEGs when $|Log_2FC|>1$ and $q<0.05$. The numbers in brackets represent the procedure number.
