# Supplementary material for: A comparison of transcriptome analysis methods with reference genome
Source: BMC Genomics. 2022 Mar 25;23:232. doi: 10.1186/s12864-022-08465-0 (PMC8957167; doi:10.1186/s12864-022-08465-0)
Supplement: Supplementary file 7 — Additional file 7. [file 12864_2022_8465_MOESM7_ESM.pdf]

Supplementary Figure 23

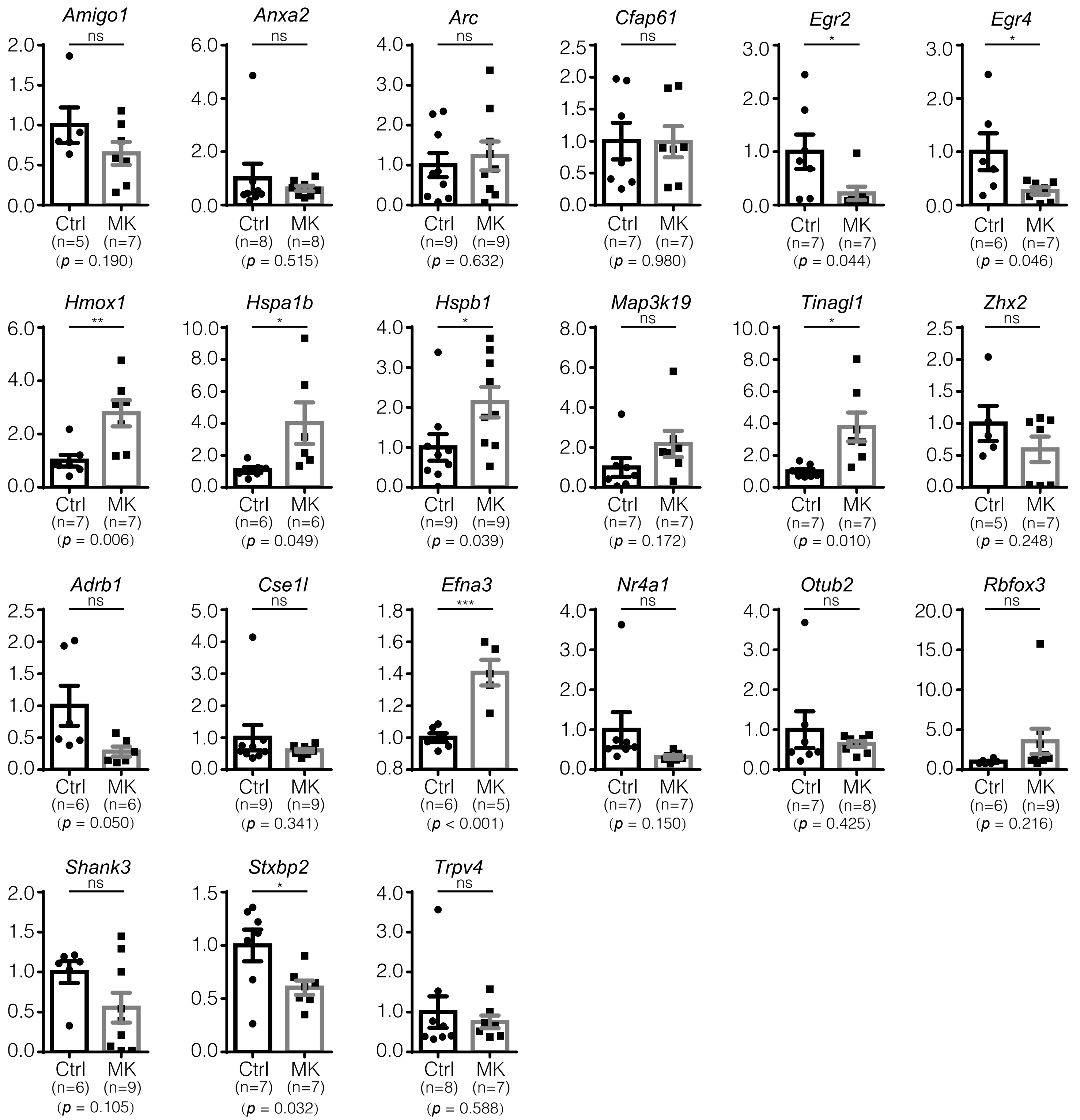

Supplementary Fig. 23 Twenty-one genes were validated via qRT-PCR experiments. These genes were differentially expressed in the results obtained from at least one procedure. Unpaired t-test, *p < 0.05; **p <0.01; ***p <0.001; ns: not significantly different.
